# Global Warming: The Balance of Evidence and Its Policy Implications

**DOI:** 10.1100/tsw.2003.26

**Published:** 2003-05-05

**Authors:** Charles Keller

**Affiliations:** University of California, Institute of Geophysics and Planetary Physics, USA

**Keywords:** climate, climate change, global warming, climate modeling, atmosphere, ocean, greenhouse gases, carbon dioxide, solar activity, environment, ecosystems

## Abstract

Global warming and attendant climate change have been controversial for at least a decade. This is largely because of its societal implications. With the recent publication of the Third Assessment Report of the United Nations’ Intergovernmental Panel on Climate Change there has been renewed interest and controversy about how certain the scientific community is of its conclusions: that humans are influencing the climate and that global temperatures will continue to rise rapidly in this century. This review attempts to update what is known and in particular what advances have been made in the past 5 years or so. It does not attempt to be comprehensive. Rather it focuses on the most controversial issues, which are actually few in number. They are: 1-Is the surface temperature record accurate or is it biased by heat from cities, etc.? 2-Is that record significantly different from past warmings such as the Medieval Warming Period? 3-Is not the sun’s increasing activity the cause of most of the warming? 4-Can we model climate and predict its future, or is it just too complex and chaotic? 5-Are there any other changes in climate other than warming, and can they be attributed to the warming?Despite continued uncertainties, the review finds affirmative answers to these questions. Of particular interest are advances that seem to explain why satellites do not see as much warming as surface instruments, how we are getting a good idea of recent paleoclimates, and why the 20 century temperature record was so complex. It makes the point that in each area new information could come to light that would change our thinking on the quantitative magnitude and timing of anthropogenic warming, but it is unlikely to alter the basic conclusions.Finally, there is a very brief discussion of the societal policy response to the scientific message, and the author comments on his 2-year email discussions with many of the world’s most outspoken critics of the anthropogenic warming hypothesis.

## INTRODUCTION

> "In the light of new evidence, and taking into account the remaining uncertainties, most of the observed warming over the last 50 years is likely to have been due to the increase in greenhouse gas concentrations." *Policy Makers' Summary, IPCC Third Assessment Report, 2001*

In 1957 noted climatologist Roger Revelle wrote: "Thus human beings are now carrying out a large-scale geophysical experiment of a kind that could not have happened in the past nor be reproduced in the future. Within a few centuries we are returning to the atmosphere and oceans the concentrated organic carbon stored in sedimentary rocks over hundreds of millions of years..."[1].

Revelle also encouraged David Keeling to make measurements of carbon dioxide ($CO_2$) levels in the atmosphere. The resulting "Keeling Curve" shows dramatically the rise of $CO_2$ above pre-industrial levels. Fig. 1 shows this rise from a combination of ice-core (pre-industrial), and atmospheric measurements as well as estimates of fossil fuel burning in recent times, which parallel the rise in $CO_2$.

Perhaps, more than anything else, this documented rise in atmospheric concentration of a powerful greenhouse gas (GHG) has served to bring the possibility of human warming of the climate to our attention. In fact, this rise makes some warming a certainty since the greenhouse effect — i.e., surface warming due to the presence of atmospheric GHGs — is real and its physics is well understood. The only questions are how much warming and how soon. The well-documented rise in global surface temperature over the past two decades has served to emphasize these questions and to focus society's interest on them.

To get the earliest possible answer to these questions the United Nations with the World Meteorological Organization formed the Intergovernmental Panel on Climate Change (IPCC) and tasked its Working Group I with:

1. Getting the best answer to the two questions above, and
2. Communicating it to policy people in terms that they can understand and use.

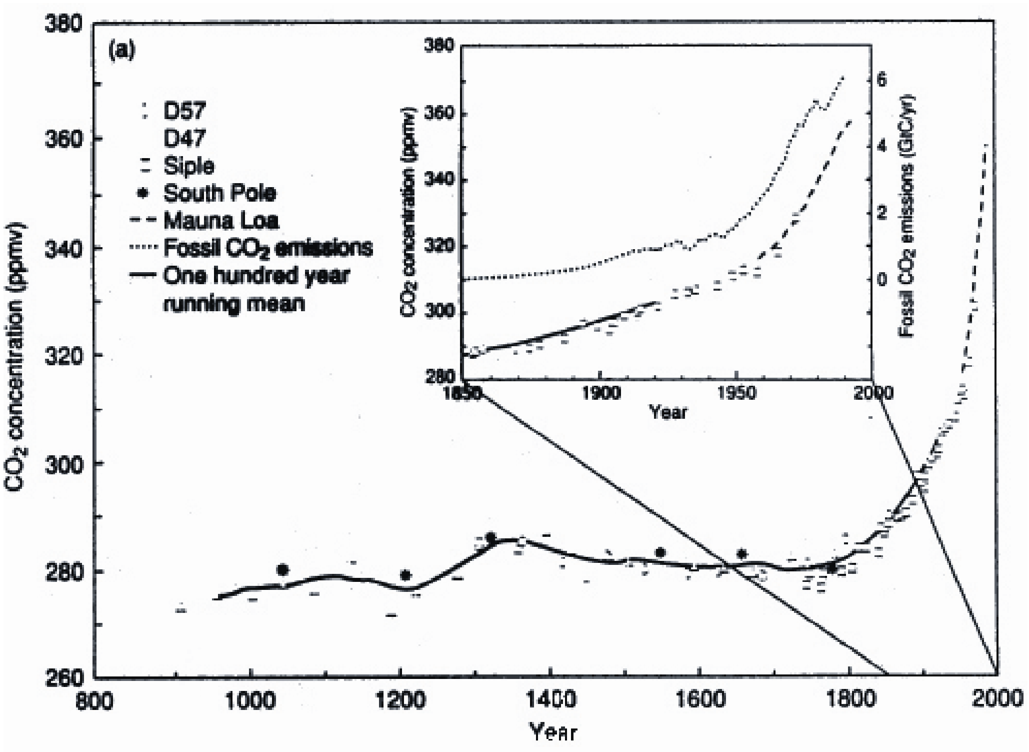

**FIGURE 1.** Atmospheric concentration of $CO_2$ compared with fossil emissions.

Most of the ensuing controversy over the answers the IPCC has been publishing probably results from the urgency associated with $CO_2$ increases in the atmosphere. Because $CO_2$ has a residence time in the atmosphere of over a century, it is important to get the earliest estimate of how much and how soon any warming might be if we are to do anything to slow projected warming before it occurs. Thus, climate scientists find themselves trying to see an initially small signal in a noisy, chaotic climate. The IPCC's 1996-very-guarded statement that we are beginning to identify this signal was an attempt to give the earliest possible warning commensurate with our understanding. But, because it is such a low confidence warning, there is plenty of room to take exception for those who think we still have not proven a human influence. This was exactly the motivation for establishment of the IPCC with its ability to provide an authoritative scientific review and to reflect a scientific consensus without undue influence from extreme positions. It is this review's reading that the IPCC has given us a fairly comprehensive assessment of the current understanding of these two questions. Summaries of the IPCC's Third Assessment Report can be found at: http://www.ipcc.ch/.

## Approach

This review of the current status of climate science applied to the issue of possible effects of increasing anthropogenic greenhouse gases (AGHG) on the climate will not attempt to be comprehensive. The subject is far too broad — witness the IPCC's Third Assessment Report, merely the first volume of which is nearly 1000 pages in length. Instead, this review will concentrate on a focused line of reasoning leading to the increasingly certain conclusion that humans are causing the Earth to warm. It will admittedly be guided in part by the objections of critics of the human warming hypothesis. These objections are along the following lines:

1. The observational record of warming at the Earth's surface is flawed and not reliable; there has been little warming since 1945 as attested to by satellite observations.
2. Climate has always varied naturally, sometimes much more than during the 20th century, and so the warming we are experiencing is probably nothing new.
3. What climate change there is can be attributed to variations in solar activity and other natural factors.
4. The AGHG villain, increases in $CO_2$, cannot reliably be attributed to human emissions given the huge reservoirs of it in the biosphere and oceans.
5. The climate is far too complex and chaotic to be modeled even by present supercomputers, thus these huge global climate models (GCMs) cannot be relied on either to attribute observed warming to AGHGs or to predict the timing or extent of future warming.

These objections will be discussed in succeeding sections. Other aspects of climate change will be summarized with references to review-type articles. The final section will touch very briefly on policy implications, given the state of our understanding.

## SURFACE VS. SATELLITE TEMPERATURES (*IS THE 20TH CENTURY WARMING REAL?*)

Of all the objections to the assertion that humans are warming the climate mainly through emissions of $CO_2$ and other anthropogenic greenhouse gases (AGHGs), perhaps the most used and discussed is the fact that temperature recording instruments aboard a continuing succession of NASA/NOAA satellites measure only a fraction of the warming observed at the surface since 1979. Some say that the disagreement between warming trends deduced from these two sources shows the surface record to be in error, i.e., the warming in the second half of the 20th century is not as large as that measured at the surface. A U.S. National Research Council panel reviewed this disagreement and concluded that very likely both records are correct[2]. We agree and will assume that both records are accurate in the following discussions. Thus the discussion will center around explaining how the surface could be warming faster than the middle troposphere measured by the satellites. Fig. 2 shows surface temperature anomalies for the past hundred years or so. The warming, however, is not smooth like the rise in AGHGs (Fig. 1). Instead there is a marked initial warming between 1920 and 1945 interrupted by a 30-year plateau whereupon warming resumes. The reasons for this will be discussed later in the modeling sections; however here we will concentrate on temperature behavior in the last 40 to 50 years. Fig. 3 shows that much of the structure in the surface temperature record is caused by El Niño/La Niña variations and by volcanoes whose eruptions are large enough to loft light-scattering aerosols into the stratosphere. It plots both global and tropics-only temperatures showing the strong influence El Niños and La Niñas have on climate.

### Surface and Satellite Temperature Records

The surface record is made from thousands of temperature-sensing installations both on land and at sea. Enormous work has gone into eliminating sources of error from this record[3]. To avoid zero point differences and similar problems unique to the site, each observing station determines its own average temperature for a prescribed number of years. Then each record is differenced from that average, generating a set of anomalies that are then combined to get average records (global, hemispheric, etc.).

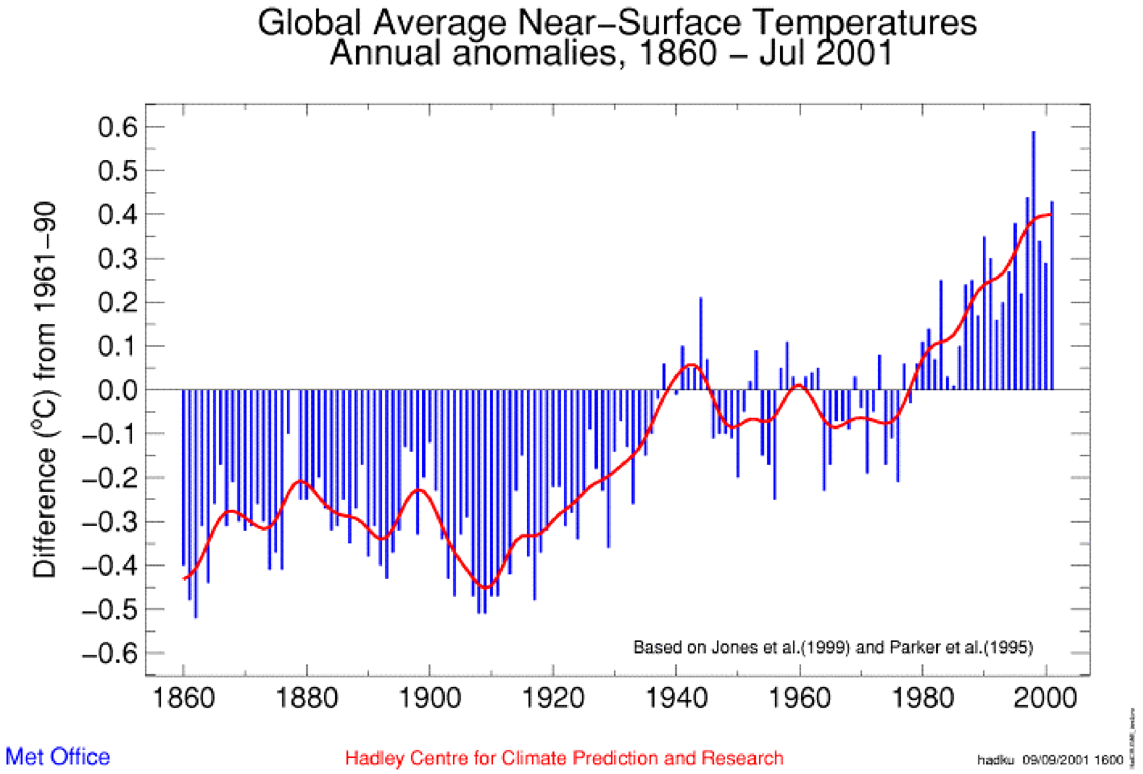

**FIGURE 2.** Global average annual surface temperature anomalies 1860–June 2001. (From UKMet Office, Hadley Centre for Climate Prediction and Research. With permission.)

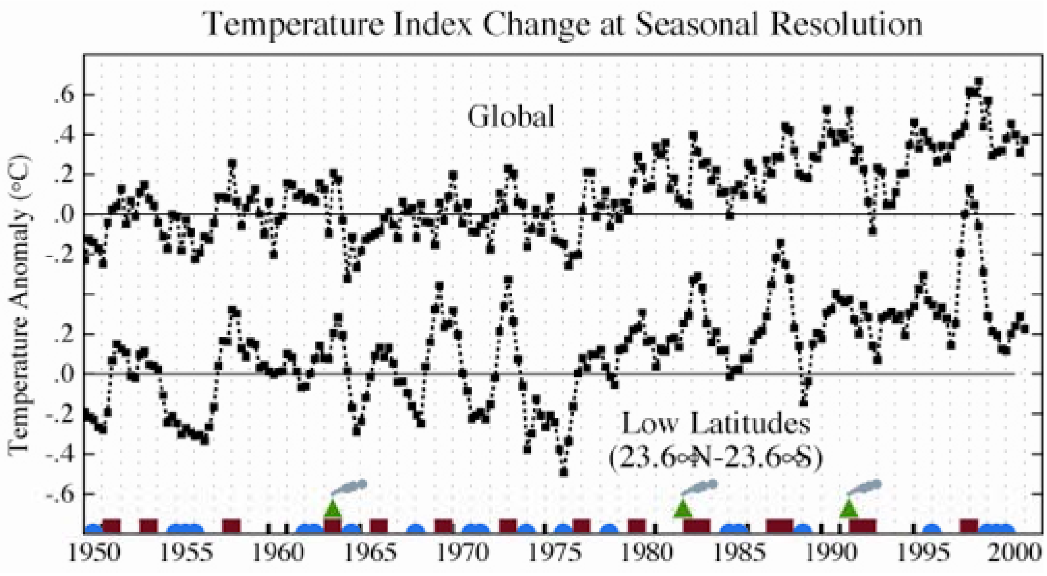

**FIGURE 3.** Global and tropical surface temperatures (1950–2000). Green triangles denote times of large volcanic eruptions; red squares-times of El Niños, and blue circles-times of La Niñas.

Perhaps the most worrisome source of error is contamination from urban areas, which usually warms more than the surrounding countryside and thus might make the warming look larger than it is. This urban heat island effect (UHI) would be expected to become increasingly important as urbanization grows. It would mimic a secular increasing temperature, particularly in the past 25 years. To avoid this problem special precautions are taken for urban data. Also several studies have been done[4,5] that compare the global temperature record between rural and urban sites. The results always come out that, for the stations used, there is not much difference between rural, suburban and urban sites — perhaps a 10% effect. Here it is interesting that the area where one might expect this effect most, Northeast United States, is actually not warming all that much. Also, surface and satellite anomalies agree fairly well over northern hemisphere (NH) land while disagreeing most over the tropical oceans (hardly an UHI effect). More recently Folland et al.[6,7] have quantified the major sources of uncertainty in the surface record. They find that between 1861 and 2000 the Earth's surface has warmed 0.61 ± 0.16°C with most of the uncertainty coming from the 1800s.

The record over the global oceans has different problems (for example, sea surface temperatures, SSTs, are used in place of marine air temperatures [MAT] — Fig. 4), and, while studies show recent disagreement between the two[8], they are not estimated to affect the general accuracy of the record[6] (see also Folland's summary of this in EOS, p. 453, Oct. 2, 2001). Finally measurements of temperatures in the deep oceans are corroborating the surface trends[9].

Satellite temperature observations are made with instruments that detect microwave radiation from oxygen in the atmospheric column (thus the name microwave sounding units — [MSUs]). These are tuned to regions of the electromagnetic spectrum that change measurably with temperature and that sample specific vertical regions of the atmosphere. Two in particular are of interest: MSU4, which measures radiation from the stratosphere, and MSU2LT from middle altitudes in the troposphere[10]. These records are fairly well substantiated by balloon observations[2,11]. From this source we now have a 21-year worldwide record of temperatures in both the stratosphere and the middle troposphere.

## *Comparing the Records*

One way to gain insight into how accurately the surface records represent global temperatures is to compare them with the satellite (MRU2LT) record. Even though the 2LT record does not measure surface temperatures, both regions of the atmosphere are expected to warm similarly. When this comparison is done, the agreement/disagreement is in the eye of the beholder. To first order the two records agree rather well (Fig. 5a). However, if a simple temperature trend is determined for each data set, they differ significantly — 0.180°C/decade for surface and 0.056°C for satellite (1979–1999). It is this disagreement that has caused skeptics to contend that, what global warming there is must be much smaller than that predicted by greenhouse gas theory since the satellite record covers the entire globe densely and is clearly without sources of surface contamination such as UHI and the SST/MAT approximation. Only part of the trend discrepancy is explained thus far[2,12,13].

Actually the two records agree fairly well over NH land areas, where both show similar large warming. It is over the tropical oceans that the two records disagree most strikingly. Finally, it has been pointed out that, even here, the disagreement is a rather recent one, largely beginning after the 1991 eruption of Mt. Pinatubo. Also a 40-year comparison of sonde (balloon) mid-troposphere temperatures with those at the surface shows both records yielding similar warming trends (Fig. 5b). Another point to keep in mind is that trends appear to vary monotonically from warming to cooling as one proceeds from the surface into the stratosphere. Thus, surface warming is about 0.18°C/decade, low/mid troposphere (MSU) is 0.06°C/decade, essentially zero in the

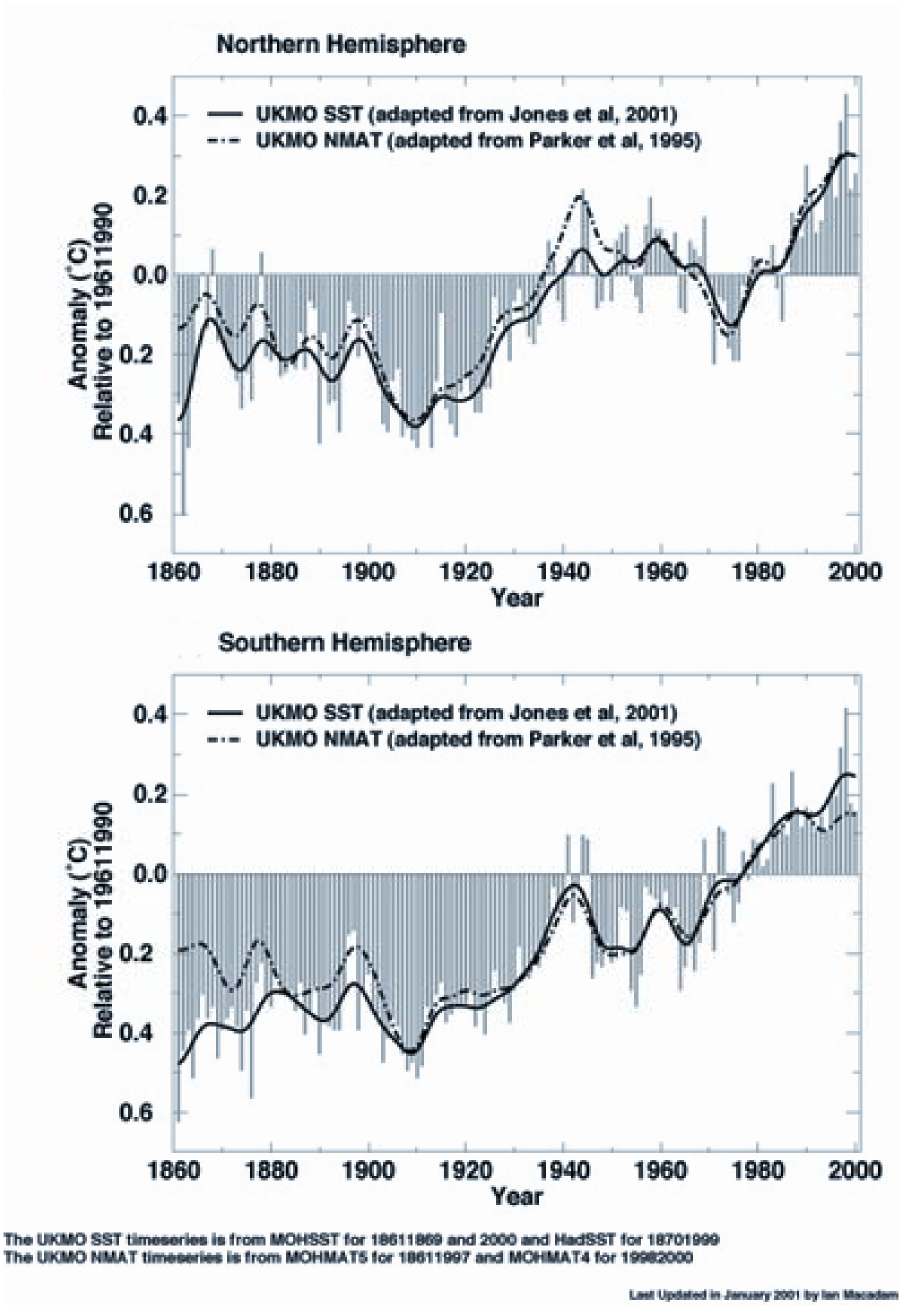

**FIGURE 4.** Comparison of sea surface temperatures (SST) with nighttime marine air temperatures (NMAT). Since NMAT measurements are difficult to obtain, SST are commonly used as proxies. Note recent disagreement 1990–2000 in the SH. (From Folland, C.K. et al. (2001) Global temperature change and its uncertainties since 1861, *Geophys. Res. Lett.,* **28,** 2621–2624. With permission.)

troposphere above that, and strongly negative, –0.5°C/decade in the lower stratosphere. This effect points to a possible explanation for the discrepancy.

The satellite record begins at the end of a dramatic rise in global temperatures measured both at the surface and from balloons (Fig. 5b). At the outset (1979–1981) the satellite record (in agreement with the sondes) showed relatively more warming than the surface record, as might be expected from Clausius-Clapeyearon effect.[1] From 1980 to 1991 satellite and surface records were

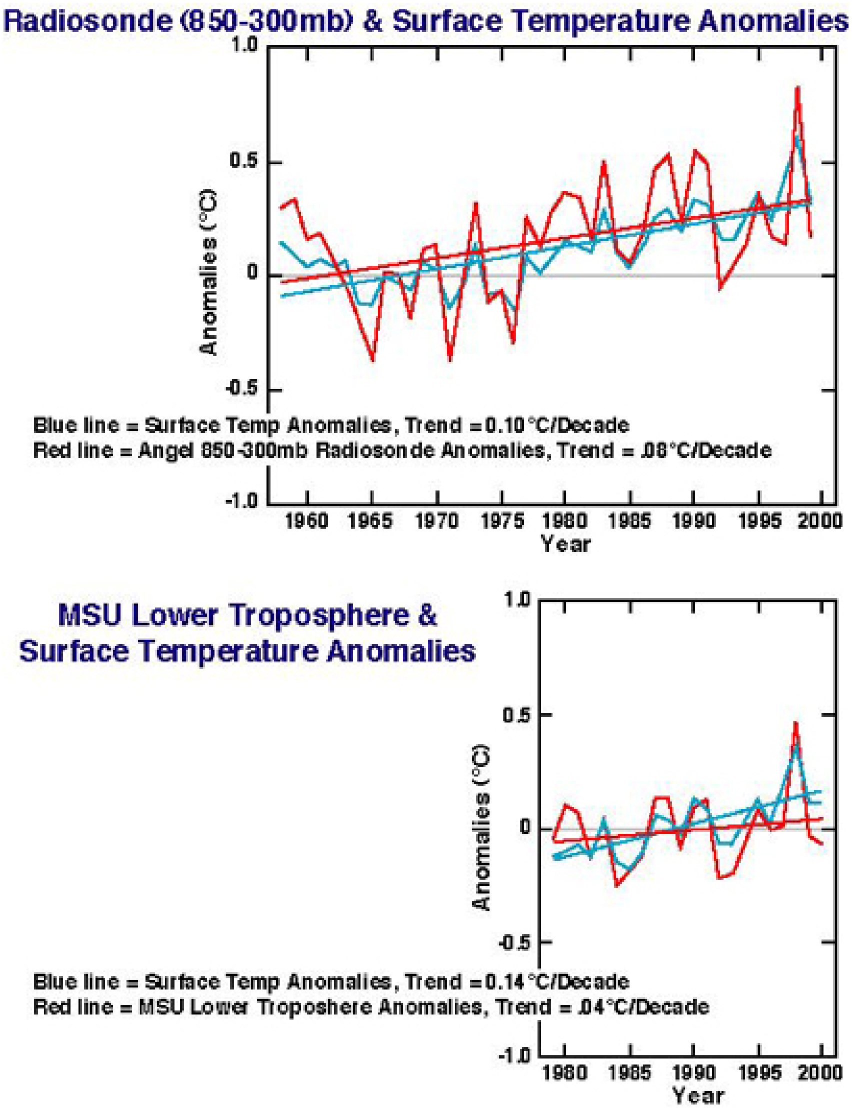

**FIGURE 5.** Comparison of yearly temperature anomalies at surface (green) and mid-troposphere (red). (a) Surface and satellite MSU 2LT for mid-troposphere (note disagreement beginning after 1991). (b) Surface and radiosonde (balloon-borne) for mid-troposphere; same effective altitude range as detected by satellites. Note general agreement of trends over this longer period. Satellite and sonde agree after 1979.

were in essential agreement through several swings of ENSO and the cooling caused by a major eruption of the volcano, El Chichon. Immediately following the massive eruption of Mt. Pinatubo (June 1991) the Earth cooled dramatically, then warmed over the next few years as sun-blocking aerosols, injected into the stratosphere by the volcano, gradually settled back into the troposphere where they were rained out. The process took about 5 years. As expected, the satellite showed more cooling than at the surface, but during the subsequent recovery period (1992–1996) it did not show its customary warming response to three successive El Niños (should have been more than at the surface).[1] Thus a difference plot of satellite minus surface temperature anomalies shows:

| | |
|---|---|
| 1979–1981 | Satellite — warmer |
| 1980–1991 | Agreement |
| 1992–1996 | Satellite — cooler |

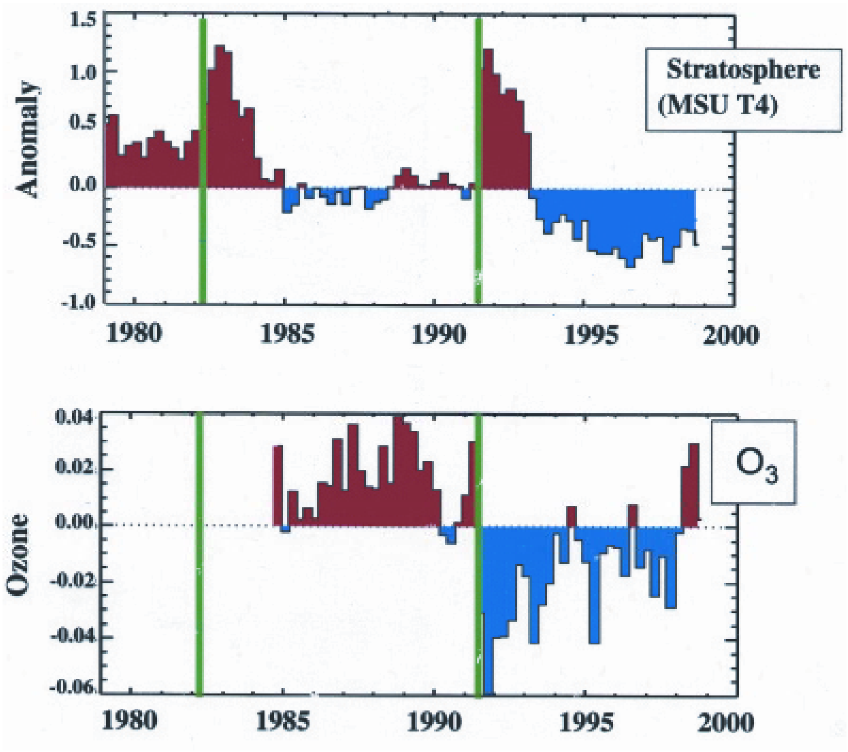

**FIGURE 6.** Comparison of lower stratospheric temperatures and ozone concentrations. (a) Temperatures; (b) ozone levels. Note stepwise drop in both records after 1991 Pinatubo eruption.

This disagreement — high early and low late — is the major reason that the satellite record shows a lower warming trend than the surface[3].

Beginning with the La Niña of 1997, the satellite once again resumed its customary response to ENSO cycles:

| | |
|---|---|
| 1997, La Niña | cooler than surface |
| 1998, El Niño | warmer than surface |
| 1999–2000, La Niña | cooler than surface |

Thus the period 1992–1996 seems anomalous when compared with behavior since 1958. It is thought that Pinatubo's sulfate aerosols may have caused this by adding to the secular reduction in stratospheric ozone (following a brief warming due to absorption of sunlight by these same aerosols, Fig. 6). This warming-followed-by-cooling behavior happened after both eruptions, but the cooling was greater after Pinatubo than El Chichon (0.3°C and 0.5°C, respectively). The cooling was very likely due to dramatic reductions in ozone caused by heterogeneous chemistry on the volcanic aerosols, adding to continued secular ozone decreases. This cooling persisted long after the aerosols settled out because ozone recovers slowly if at all. The dramatically cooled lower stratosphere in turn had a cooling effect on the upper and middle troposphere where the satellite was measuring temperature. This would explain both the lower warming trend and also the failure of the mid-troposphere to respond to El Niño warming. In fact the lower stratosphere continues very cold up to the present, which may be causing a cool bias in the troposphere.

There is further confirmation of this rationale. The lapse rate (the rate at which the troposphere cools with altitude) has been observed to be changing in response to some forcing mechanism. Two recent papers[14,15] have looked at lapse rates in the tropics. (In addition, I recommend the "Perspectives" article by David Parker in the same *Science* issue as the Gaffen paper. It is a good statement of where we are with this problem.) In the first paper the authors use radiosondes — balloon-borne instrument packages — to measure both the surface and tropospheric warming. In the time period 1960–1997, they find that, in relative agreement with computer codes, the middle and upper troposphere warm at a rate of 0.19°C/decade, more than the measured warming at the surface, 0.12°C/decade, but during the time period of satellite observations (1979–1997) the trends are reversed with mid-upper troposphere cooling at the rate of –0.10°C/decade while the surface trend remains the same. The authors conclude "temperature and lapse-rate trends during the MSU (*satellite*) period are qualitatively different from the preceding two decades." The second paper concurs and concludes: "This lapse rate variability could account for a significant portion of the difference between the observed surface and the lower tropospheric temperature trends, as measured by MSU and radio sondes, since 1979." Thus, both papers agree that in the topics *the differences between warming at the surface and at MSU effective heights are real*. They further agree that this is largely caused by marked changes in lapse rates. And finally, when a longer period than that of the MSU record is looked at, the warming trends, surface and troposphere, are about the same.[2]

Finally, and this might be significant, both papers agree that computer simulations are unable to reproduce this lapse rate variability. This is probably due to some shortcoming in the models. Apparently some other mechanism or forcing is operating that is not included in the models. Several come to mind — some effect of solar activity that acts only high in the troposphere[16], or, in the case of the discrepancy after 1992 — a cool upper boundary condition due to marked tropospheric cooling following Mt. Pinatubo-induced ozone destruction[17,18], or some lower boundary condition such as too simple an ocean model. These possibilities will be discussed in the modeling section below.

Another approach to compare the surface and satellite records is to remove the large interannual variability due to ENSO and volcanic eruptions. Several attempts have been made[19,20,21]. Two find little warming after removal, but the third finds very large warming. Perhaps the most comprehensive effort is by a large team[22]. This last effort shows a range of warming from both surface and satellite (0.210 to 0.250°C/decade and 0.056 to 0.158° C/decade, respectively). Also the authors suggest that this means the effects of large ENSO and volcanoes cool the troposphere more than the surface as suggested above: "Had Pinatubo and El Chichon not occurred, it is likely that the lower troposphere would have experienced more pronounced warming." They perform the same separations on several climate modeling results and show that at least part of the discrepancy in warming trends is being addressed in the models (more recent models in the sections, "Computer Simulation" and "Attribution and What Might We Expect in the Future" further close this gap).

## Summary

The evidence supports the NRC report's conclusion that both the surface and satellite (tropospheric) records are substantially correct. At issue is the accuracy of the surface record, i.e., where humans live. The reason for the difference in temperature trends between the two remains only partially explained; however, possible errors in the surface record from urban heat island and sea surface temperature approximation of marine air temperatures do not appear large enough to explain the discrepancy in recent warming trends. In attempting to understand the difference between the two, we are realizing the importance of correctly simulating the lower stratosphere and perhaps the oceans for correct modeling of tropospheric temperatures.

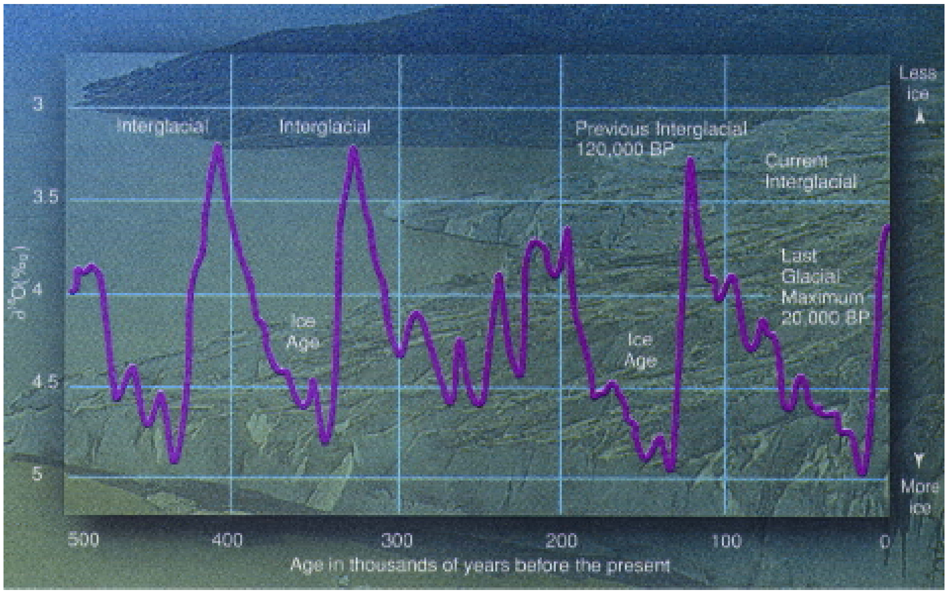

**FIGURE 7.** Variation in mass of global ice in continental ice sheets from marine sediment oxygen isotope records. Note plot is upside down to show warm periods at top and cold at bottom. (Taken from NOAA reports to the Nation, No. 4, Fall 1997.)

## CLIMATE VARIATIONS (IS THE 20$^{TH}$ CENTURY WARMING SPECIAL?)

If the 20$^{th}$ century warming is real, it may still be argued that it is not "special", i.e., there have been other warming periods as great or perhaps greater. To answer this question we consider what is known about the Earth's temperature before the instrumental record began in the middle of the 19$^{th}$ century. To be sure, climate has varied considerably over the age of the Earth; however, for most of that time conditions were so different as to make those climates not good analogues for today's situation. Over the past million years or so the climate has alternated between glacial and interglacial periods. These alternations are thought to result from three cyclic changes in the Earth's orbit and orientation toward the sun. The record from the Antarctic Vostok ice core shows at least five ~135,000-year-long glacial/interglacial cycles (Fig. 7). The question of what caused these oscillations between cold and warm periods was answered in large measure by V. Milankovitch, who noticed that changes in the Earth's orbit and aspect toward the sun could explain the broad features. There are three such changes:

the shape of the Earth's orbit around the sun: eccentricity
the tilt of the Earth's rotation axis: obliquity
the "wobble" of the Earth's axis: precession

These changes cycle completely in about 100,000, 44,000 and 23,000 years, respectively, and the interplay of the three causes a complicated pattern of the amount of sunlight reaching the NH. If the land and oceans were divided symmetrically between NH and SH, these variations would also occur symmetrically and would have little effect. But most of the land mass is in the NH, and, since it responds with a larger temperature amplitude to changes in sunlight, these cycles can have marked climate change implications. When one combines variations in sunlight due to these three effects and compares their combined effect with the Vostok core's record of amount of ice covering the land, the two look very much alike, suggesting that Milankovitch cycles initiate major climate changes over at least the past half million years. However, the cycles must be amplified by some additional feedbacks since the amount of sunlight variation they cause is not nearly large enough to force the observed changes in global temperature. Recent modeling shows that a combination of variations in $CO_2$ and ice cover can provide the necessary amplification. While researchers still differ on exact timing of $CO_2$ forcing relative to paleo-temperature response to Milankovitch cycles, recent work by Shackleton[25] and others suggests that $CO_2$ changes preceded temperature change. However, even if the changes were much closer in time, the picture still makes sense with $CO_2$ and ice acting as positive feedbacks.

## The Last 10,000 Years

Global temperatures reached a maximum sometime in the early Holocene (about 10,000 years ago) and have been declining slowly ever since $wi_2$changes in the precessional cycle. Fig. 8[26] shows this effect via three independent proxies of temperature:

ice melt in core from Ellsmere Is.
elevation of treeline (Sweden)
oxygen isotope temperatures derived from stalagmites (Norway)

These agree on a secular decline in atmospheric temperatures, and it is against this backdrop that we look for additional, shorter-scale climate variation. What we find in the last 1,500 years is an alternation between warming and cooling characterized by the two most significant events, the Medieval Warming Period (MWP) and the Little Ice Age LIA).

The MWP occurred between 1000 and 1300 when Europe and the North Atlantic were relatively warm. The LIA followed between 1500 and 1900, when they were abnormally cold. Indeed, if we can believe historic records from Europe, which told of warm weather crops' being grown far north of where they grow now, the MWP for Europe must have been as warm or warmer than at present. But was this warming and subsequent cooling global or even hemispheric at such large amplitude? The best answer with the sparse data we have is maybe, but probably not.

While the LIA seems to have been global in extent, the MWP appears not to have been. The MWP from different records seems spread out over two or three centuries, occurring at different times in different places, but not generally if at all in the SH[27]. One multiproxy reconstruction[28] shows that the peak warming seems to have occurred in the 12$^{th}$ century. Other records suggest it occurred earlier. The LIA also seems to have been a multi-episodic event generally of the late 17$^{th}$ and mid 19$^{th}$ centuries. It is the regional timing of these events that makes them hard to pin down. For the same reason, when the best proxy records from tree rings, ice cores, historical records, sediments, etc. are averaged, many features cancel each other out and the resulting curve shows a much calmer, low amplitude record on average. On the other hand these records all agree on one thing, the rapid rise in temperature in the 20$^{th}$ century. Also, there seems to have been less spatial and temporal variability in climate change in the last century than in earlier times. And it is the homogeneity of the 20$^{th}$ century record (as well as its rapid rise) that separates it from these other events, not just its amplitude. But how are these variations determined and how accurately?

## Temperature Proxies

Temperature proxies — that is, measurements that provide indirect indications of temperature— exist for many localities and regions back to the 16$^{th}$ century, but high quality/resolution records back 1,000 years are few. Nevertheless several groups have attempted to determine hemispheric temperatures back that far from an ensemble of regional records, selected to be representative.

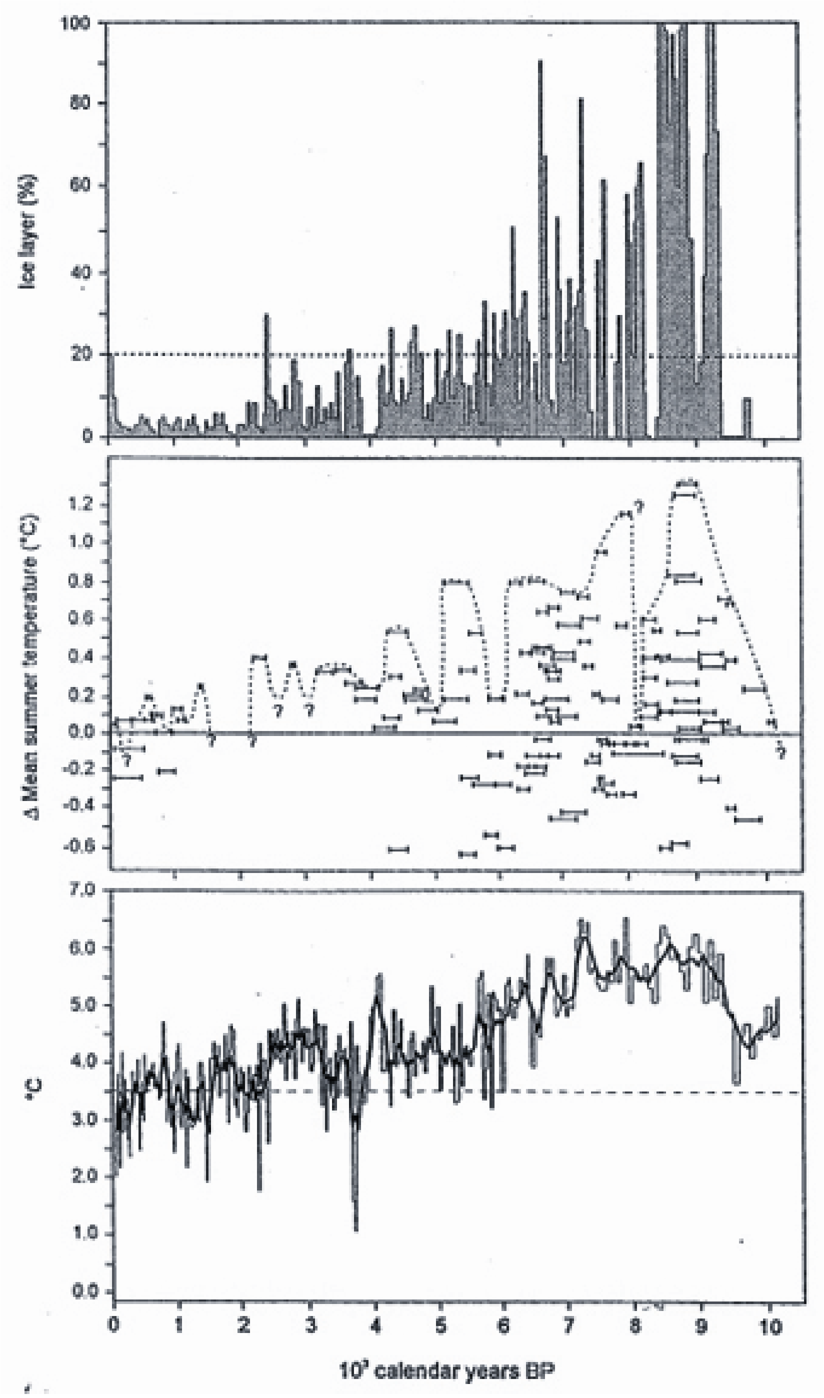

**FIGURE 8.** Temperature indicators during the present interglacial (Holocene) from (a) ice melt in core from Ellsmere Is., (b) elevation of tree line (Sweden), and (c) oxygen isotope temperature proxy from stalagmites (Norway).

Two excellent summaries of this work to date are found in Jones et al.[29,30]. They discuss the problems involved and consider the multiproxy compilations published since 1998[28,31,32,33,34]. Finally I recommend an excellent short summary by Bradley[35].
The temperature proxies most in use are tree rings
ice cores
ocean and lake sediments
corals
stalagmites
historical records
altitude of treeline

A good introduction to how these are used is given by Erik Stokstad[36]. A more detailed treatment is Bradley's book[37]. Circumstantial evidence comes from archeology and other sources, but the above can be quantified more reliably. Even so, each has its problems largely because temperature is not the only cause of the variations in these records. Old age in trees, growth factors in corals, deposition in sediments, rates of snowfall in ice cores, etc. all contribute to uncertainty in temperatures. Considerable effort thus goes into accounting for these problems[38,39].

## The Last Thousand Years

There is a plethora of individual records[40,41,42,43,44,45a,45b]. Of particular interest is the temperature amplitude between the peak of the MWP and the LIA minima. Depending where and how you look, this amplitude varies from very small to rather large, mostly in the range 0.3° to 2.0°C. But to compare past climates with the last century, one needs some global or at least hemispheric average of these records. Several such compilations have been done involving a mix

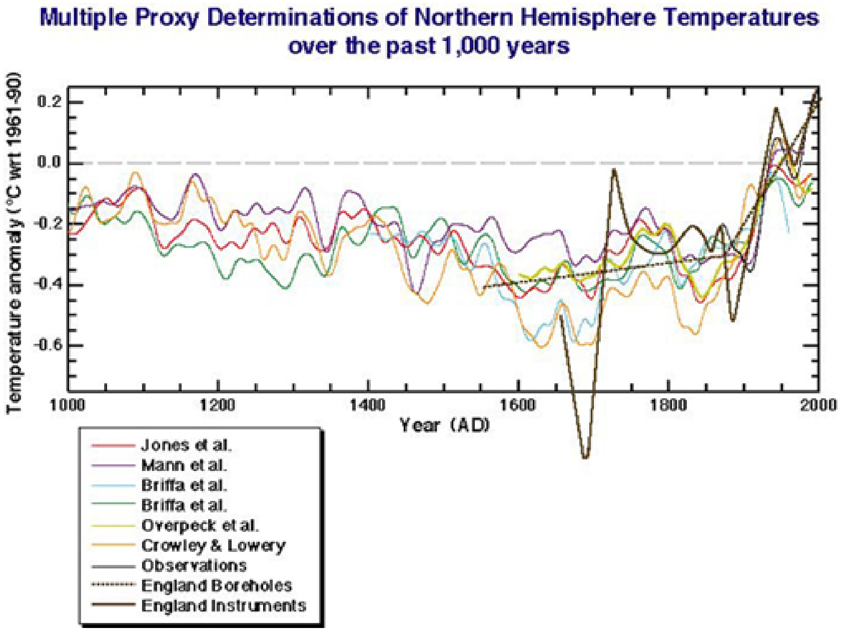

**FIGURE 9.** Most recent temperature proxy compilations for the NH. Also plotted for comparison are average borehole and instrumental temperatures for central England.

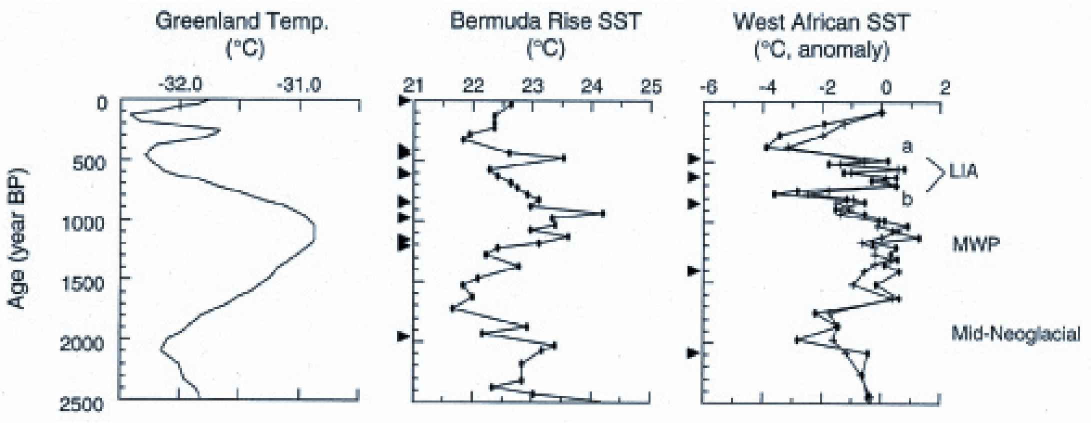

**FIGURE 12.** Examples of three large amplitude temperature records. Note low frequency variations agree on warming around the year 1000, but disagree in detail after 1500.

of the above proxies. Fig. 9 shows these. Note that the total amplitude indicated is <0.6°C. However, in contrast (Fig. 10), some individual records show much larger changes such as from an ocean sediment record[41], borehole records in the Greenland ice cap[44] and West African sea temperatures[42].

The situation is further complicated by introduction of a nonproxy actual temperature record — thermometry from inside boreholes. This method determines paleo-temperatures by inverting observed temperatures measured within boreholes, either in ice or ground. This is quite an independent method and has much promise. In general it gives similar results to those of other proxies, but in detail it differs in important ways. When records from hundreds of boreholes around the world are compared with results from other proxy data, two things are noticed. First, the borehole results are very smooth and show only long-term, low frequency trends. Second, the borehole results usually show a larger, long-term total temperature amplitude than proxy compilations[46]. This difference in temperature amplitude between borehole and proxy data has been cause for some concern as to which is correct. Given the difficulty of some of the proxy data in showing low frequency variability one is tempted to rely on the borehole results. But they, too, can be biased because of surface changes at the borehole such as land use (clearing, etc.), which would make earlier temperatures look cooler than they actually were.

Indeed Jones and Briffa[29] might be teasing out just this effect. They show an extended instrumental record from England going back to the late 1600s and another for Central Europe from 1750. It agrees with the borehole record for that region. Curiously, while the global borehole record indicates a total temperature variation amplitude of about 1.1°C between 1600 and 1945, the England and Central Europe borehole records suggest an amplitude of ~0.4°C, in agreement with both the instrumental record and that determined from proxy reconstructions calibrated to instrumental records (Fig. 9). Since in England and Europe land use has not changed much in centuries, might the records from those areas not be more accurate than the global one, which has experienced large, recent surface changes? Countering this attractive rationale are two borehole thermometry records from the Greenland icecap[44], which show a long and large amplitude MWP and a relatively deep LIA (which correlates rather well in timing and amplitude with a Sargasso Sea sediment $\delta^{18}O$ record[41] and Fig. 10). To further the confusion, temperatures inferred from oxygen isotopes taken from the ice core at the GISP Greenland site show no substantial MWP and only a modest LIA. Thus, on the same ice sheet, proxy and thermometry methods disagree. Given these problems, some are tempted to rely only on boreholes and altitude of treeline for multicentury temperature reconstructions[47]. It will require more study to unravel these conundra. For the present, perhaps the best approach is to continue to compare borehole thermometry with other types of records and to assume that the real answer is probably somewhere between the two.

One point, however, is becoming increasingly clear. Past climate seems to have been spatially more heterogeneous than recently. Intercomparison of proxy records shows that they disagree as often as agree. The picture they present is a hemispheric/global climate that is a composite of very differing regional temperature histories. This, of course, could simply be defining the level of accuracy (or inaccuracy) of the methods; but if it is real, it suggests that earlier climate, forced only by natural events, was more regionally variable than in the last century or so. This could mean two things:

1. Determining a truly hemispheric or global paleo-temperature record will require a very carefully selected set of records.
2. Climate variations before the 20$^{th}$ century were not as regionally uniform as recently.

This in itself could be a defining characteristic of GHG warming. As one noted paleoclimate researcher remarked:

"The 20$^{th}$ century warming, and 19$^{th}$ century cooling are the most extreme and coherent trends common to the northern sites we have studied. These recent, more coherent trends suggest the possibility of stronger common forcings, becoming more dominant over regional variations, in particular the increasing trace gases in the 20$^{th}$ century. Less coherent fluctuations among the records back in time may signify more regional effects, and also the need for additional coverage for a more accurate perspective of climatic change"[43].

## Summary

Paleoclimate prior to the last thousand years or so has been influenced by factors not relevant since. Attempts to determine recent paleo-temperatures are complicated by large uncertainties. With these cautions in mind we can still say that temperatures in the past 1,000 years or so are becoming determined well enough to be compared with the recent warming. The picture is that of a slow, secular cooling throughout the Holocene related to precession. Superimposed on this are solar activity variations that produce significant variation in the signal. Most recently is a slow warming, MWP (at least in the NH), beginning about 1,500 years ago and continuing sporadically, varying from place to place and time to time till about 1200. Conversely, the 16$^{th}$ century saw cooling that deepened and lasted through the 17$^{th}$ century. The relatively warm 18$^{th}$ century saw a reversal in temperatures that lasted into the 19$^{th}$ century when the earlier levels of cooling returned and lasted until the early 20$^{th}$ century. Thus the so-called LIA was really two or more episodes with warmings in between. Hemispheric and global warming in the 20$^{th}$ century appears to have been larger, more rapid and more uniform than any time in nearly 2,000 years (although certain years or even several year periods were quite warm and in some regions that warming probably equaled that of the 20$^{th}$ century.

In terms of the debate, determination of temperatures in the past 1,000 years reduces to essentially one disagreement. Paleoclimatologists for the most part reconstruct past temperatures by averaging proxy records. Because warmings and coolings are often nonsynchronous, this procedure generates relatively low amplitude variations (0.4–1.1°C). Critics are impressed instead by the amplitudes of temperature variations in individual records and thus they average, not the records, but the amplitudes. This results in significantly larger amplitudes (1.0–2.0°C). Regardless, if the averaged amplitude for the last 1,000 years is close to the upper limit (greater than 1°C), natural forcing will have to be reassessed since solar alone would then be big enough to account for almost all 20$^{th}$ century warming. If, on the other hand, and this appears to be the case, the amplitudes turn out to be less than 0.7°C and other forcings such as volcanoes provided a serendipitous negative forcing[48,49], then solar forcing becomes smaller, accounting for less than half of 20$^{th}$ century forcing. Finally, here it could be objected that other sources of natural variation might have been involved in a significant way in determining climate over this time period. It is hard to answer this objection because such unidentified variation might take the climate in either direction. In the absence of indication that additional substantial variations may be present, the community has made the working assumption that if present, they are minor.

## SOLAR FORCING OF CLIMATE (ARE INDIRECT SOLAR EFFECTS AFFECTING CLIMATE?)

A common alternative explanation to anthropogenic greenhouse gas climate forcing in the 20$^{th}$ century is that our Sun is responsible for observed temperature increases. Have not there been significant temperature variations during the Holocene (last 10,000 years) when there were no fossil fuels being burned?[3] If the Sun's variability caused those, why could not it be causing the present warming? Perhaps a more focused question would be: "How much of the observed warming in the 20$^{th}$ century is caused by changes in solar activity?"

Much work has gone into looking for correlations between northern hemisphere (NH) temperature variations and proxy determinations of solar variability (because land surface, which predominates in the NH, responds to solar variation more than sea surface).[3] There seems no other way to explain such historical events as warming in medieval times and cooling in both the 17$^{th}$ and 19$^{th}$ Centuries excepting by solar variations and resulting feedbacks (with the complication of assorted large volcanic eruptions)[49].

The Sun is a variable star. In fact other stars like our Sun are seen to vary also[50]. In a cycle that varies in length from 10 to 12 years, sunspots come and go, the solar wind strengthens and weakens, and other associated phenomena are observed changing. Also the longer-term level of solar activity changes with time — now getting stronger with more sunspots, now getting weaker with fewer (see sunspot numbers plotted in Fig. 12). In fact in the 1600s there were no sunspots at all! This has been called the Maunder Minimum and occurred at roughly the same time as the so-called "Little Ice Age" when temperatures around the planet were cooler than normal.

If one looks at the record of sunspot numbers vs. time, the 10- to 12-year cycle is obvious, but also obvious is that the maximum number of sunspots in each cycle varies. These appear loosely to be modulated by an 80 year envelope, called the Gleissberg Cycle.[4] Comparison of the ups and downs at the maximum of each activity cycle with NH temperature estimates over the past few hundred years shows a correlation, but it is not strong largely because at times it lags the temperature changes by up to two decades. However, another correlation was found that was much better. Instead of correlating with activity amplitude, Friss-Christensen and Lassen[51a,b] correlated NH temperature change with the *length* of the solar cycles. Surprisingly, this gave a very good correlation, particularly because its ups and downs in the 20$^{th}$ century were some 20 years on average ahead of a similar pattern found in the solar activity amplitude record, allowing it to coincide with temperature changes. Extension of this proxy back to 1550 seemed to give equally good results (Fig. 11a) — but another attempt to repeat this work arrived at less good correlations (Fig. 11b).

To give these correlations a more physical basis, all that was needed was a quantitative measure of irradiance change with solar activity, because, unless solar irradiance was varying with solar activity, there was no accepted mechanism for how these changes could affect Earth's climate. Satellites began measuring the solar flux in 1979, and so by the early 1990s we had those numbers.[5] Armed with this 20-year quantitative measure, several groups attempted to calibrate the changes in solar irradiance in the past few hundred years. Most quoted are two works: (1) Hoyt and Schatten[52] reasoned that changes in convective activity were the root cause of

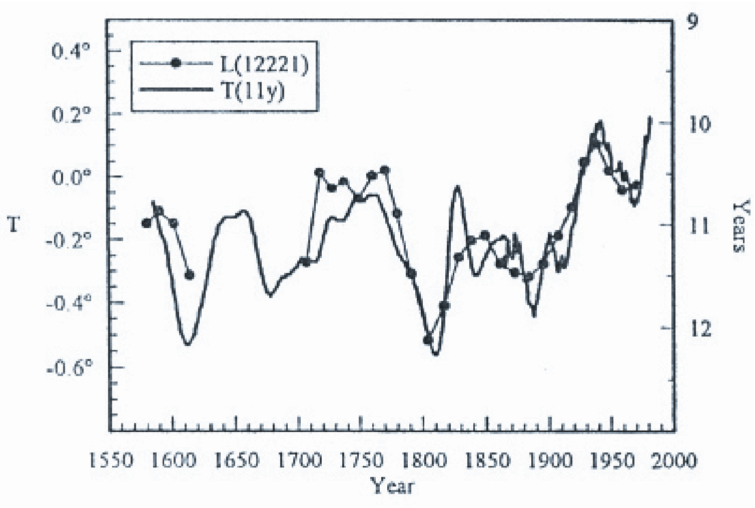

**FIGURE 11.** (a) Comparison of length of solar activity cycle with global temperatures[51b]. L is the filtered value (1/2/2/2/1 filter) of sunspot cycle length, T is the 11-year running average of Northern Hemisphere temperatures from Groveman and Landsbert [130] and Jones [131]. (b) Similar to (a) — sunpot cycle length (circles), but calibrated to more recent temperature reconstruction (solid line). Instrumental temperature record included for comparison (dots)[55].

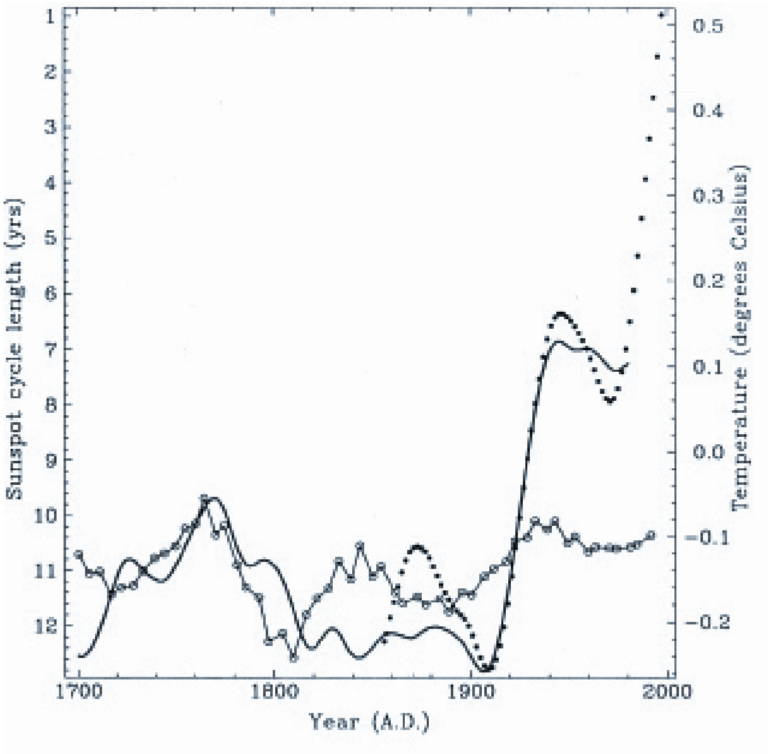

**FIGURE 11B**

changes in the length of the solar cycle. They related irradiance changes to this and developed a proxy record based on length of the cycles that could be followed back to 1700. Lean et al.[53] developed a similar reconstruction but based on activity amplitude of the solar cycles that could be traced back to 1610 (Fig. 12).

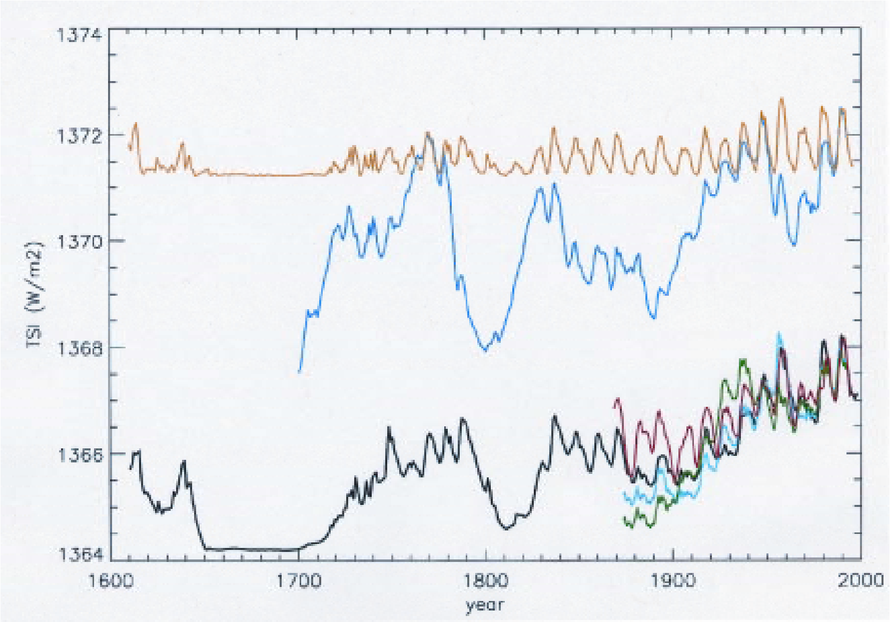

**FIGURE 12.** Proxy reconstructions of solar irradiance from two methods: blue[52] and black[53] with two more recent determinations and assumed forcing simply due to sunspot numbers calibrated with satellite irradiance observations from the past 20 years.

To assess how much of the proxy temperature variations over this time interval could be attributed to solar activity variation, energy balance models using these proxy irradiance reconstructions and assuming feedback mechanisms similar to those found for GHG forcing were employed. These showed that solar variations could account for most of the NH temperature changes until midway in the 20th century. They suggested that increases in solar irradiance could

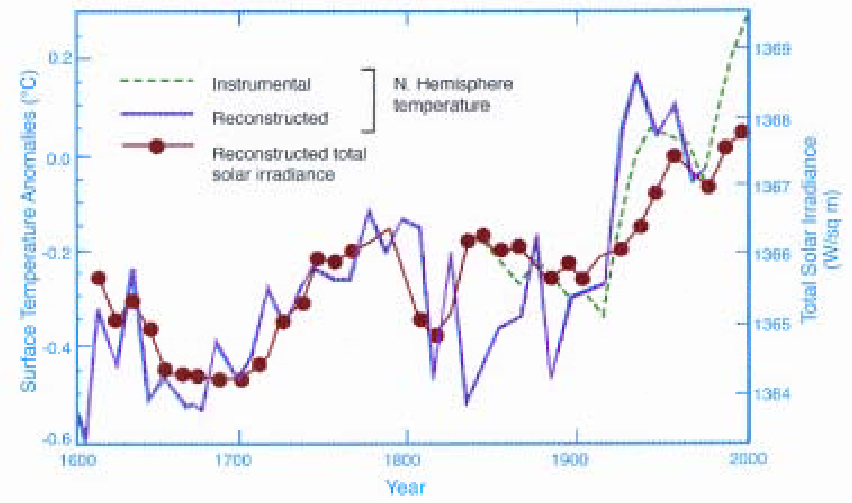

**FIGURE 13.** Comparison of observed surface temperature variation with that derived from models using proxy solar forcing. (From Lean et al. (1995) Reconstruction of solar irradiance since 1610: Implications for climate change, *Geophys. Res. Lett..*, **22,** 3195–3198. With permission.)

account for 30 to 50% of the 20th century warming (Fig. 13). Some attempts to reproduce these results with better models have tended to side with the lower part of this range[54], and recent studies[48,55] and suggest only about 25%. (These included the cooling effects of volcanic eruptions and used the most recent temperature reconstructions [Fig. 9] which show less temperature variation than earlier work.) However, other recent detailed studies using both H&S

and Lean et al. forcing[56,57,58] show that, in order to explain the 20th century record well, one needs 30 to 45% solar and the rest a combination of AGHG positive forcing and aerosol negative forcing.

So the answer to our original question is that, yes, some of the warming in the 20th century was caused by increases in solar activity–related irradiance, but not all, apparently not even half. See the recent excellent summary article on this by Lean and Rind[59].

## Possible Larger Indirect Solar Forcing

Largely because direct (irradiance) solar forcing is unable to explain the 20th century NH temperature surface record, and because many think temperature amplitude in the last 1,000 years was fairly high, it has been suggested that there is additional forcing from solar activity variations due to some indirect, solar-related amplification mechanism. Several have been proposed[60,61], but most center around two mechanisms:

1. Changes in the strength of the solar wind
2. Changes in the Sun's ultraviolet (UV) radiation

In the first of these, the proposed mechanism is the known modulation of cosmic rays by the solar wind. During high/low solar activity, fewer/more cosmic rays get to the Earth due to stronger/weaker solar wind intercepting them. It is suggested that cosmic rays provide cloud condensation nuclei for condensing water vapor. Thus, during high solar activity, fewer cosmic rays would mean less cloudiness/more sunlight and a warming would occur. Conversely, during low solar activity the reverse would cause cooling. If this were so, the reasoning goes, global cloudiness should correlate well with changes in the solar wind strength. Svensmark and colleagues have published papers showing this correlation using satellite-determined cloudiness[62,63]. However others[64,65] do not concur. When they look at the record, they do not get a very good correlation between cosmic rays and clouds. There has arisen a back and forth exchange on what is the best data set, what is the best way to sample it, what are the problems with it, etc. There are two points to keep in mind here:

1. Cloudiness seems to be in the eye of the beholder.
2. Cloudiness is not climate change. There must be a concomitant global temperature change.

There are basically two records. One is from ground-based observations of cloudiness over the past 50 years or more. These records see low clouds, an important point since these are perhaps the most important in cooling the planet. But this means that often upper level clouds cannot be seen. Also it is clear that ground-based observers sample but a small part of global cloudiness. This record shows no correlation with solar activity. Satellite records are more comprehensive in coverage, but these observations are a mere 20 years old (~2 solar activity cycles). Further, Norris[66] points out that there seems to be an observational selection factor determined by what areas the stationary satellite observes most. A third curious observation adds to this discussion. By observing Earthshine reflected from the New Moon one can watch the Earth's albedo change with time. Early results seem to show that the albedo changes along with the solar cycle as the cloudiness mechanism[67] suggests. This would seem to support the solar indirect forcing hypothesis, but it says nothing about point #2 above, and the solar cloudiness forcing mechanism has been questioned on the grounds that, if it were large enough to account for all the 20th century warming, its effects would be seen as a marked temperature variation over the Sun's 11-year activity cycle (point # 2). Arguments that the ocean's heat capacity would induce a lag in the climate system ignore observations that the Earth's temperature has indeed been observed to vary over the solar cycle albeit with very low amplitude commensurate with variations in direct solar forcing. Indeed, White et al.[68] showed that the very global ocean, which is supposed to cause a lag in temperature, actually is observed to have heat variations in its upper layers in sync with the solar cycle. However, these variations are small and can be largely explained by solar direct forcing, which is too weak to cause the observed secular warming. Still, some solar indirect forcing may be present. (Recently they have suggested that a small solar indirect forcing may be ordering natural modes of variability of the Pacific Ocean[69].) Others come to much the same conclusion[70,71] of very small indirect effect. However, even if some indirect (cloudiness) forcing exists, solar activity peaked around 1960 and so it is unlikely to add much to warming since then.

The second proposed mechanism for solar indirect forcing is changes in stratospheric ozone due to large ultraviolet solar radiation changes over a solar cycle[72,73] . While the Sun's total irradiance change is small over a solar activity cycle (~0.15%), its UV radiation changes by some 10%, strongly affecting ozone abundance in the stratosphere. Since ozone is a greenhouse gas, this should affect global temperatures. Labitze and Van Loon[72] observed changes on alternating cycles of the upper atmosphere's quasi-biennial oscillation, and Haigh[74] and Shindell et al.[75] have attempted to model the resulting affect. It appears to be present but again small. Finally, Udelhofen and Cess[76] found a positive correlation (0.71) of U.S. cloud cover with solar activity, but not with the variations of cosmic galactic rays as Svensmark has suggested, which should be anticorrelated. They also found that cloud cover simulated in a coupled climate model[109] was correlated with solar activity. However, since only solar direct forcing was employed, they concluded that the modeled variations were due to the modulation of UV radiation as suggested above. Other modeling studies are able to reproduce multidecadal global temperature observations without recourse to the Svensmark mechanism[58,108]. There remains the possibility that improved computer models will show that so[illegible]olar indirect forcing is necessary and why the 11-year solar activity cycle is not more evident in the temperature data.

## Summary

The magnitude of solar forcing varies with the amount of solar activity. In addition to the 10- to 12-year variations, solar maxima vary over multidecadal periods. Of particular interest were the Maunder Minimum, when solar activity was minimal for several decades, and the sharp multidecadal increase in solar maxima during the first half of the 20th century. Satellite observations of solar irradiance for the past two decades have allowed calibration of variations in solar activity. A number of attempts at reconstructing past climate variations via solar forcing have shown that, prior to 1940, direct forcing by changes in solar irradiance can explain most of these changes, but these have been unable to account for warming since 1975. Indirect solar forcing effects seem to be small, but merit continued study.

## COMPUTER SIMULATIONS (HOW GOOD ARE THESE ANYWAY?)

GCMs integrate physical processes that regulate the Earth's climate by coupling models of the atmosphere, land, oceans and sea ice. The climate system is so complex that without these models it is difficult to make sense of the myriad of data on the various aspects of how the Earth handles its heat budget from incoming sunlight to outgoing longwave radiation (OLR).[6] Consequently, these models are very useful in guiding the intuition of researchers and have taken their place as indispensable in the ongoing attempt to understand how our world works. This was an extremely useful role when climate was viewed as something totally natural to be studied. But with the advent of the possibility that humans are changing the climate, this role has expanded as policymakers seek advice on the magnitude of changes and how to deal with them. GCMs now carry this heavier burden because their performance can affect entire changes in societal actions and have large economic and social consequences. The question is, just how well can one expect to simulate the chaotic, complex processes that go into making up climate. Is it even possible? What can we reasonably expect the models to tell us? What will they never be able to simulate? Can they be relied upon to predict climate?

## Simulation of Present Climate

The first step in answering these questions is to compare model results with observations of the present climate to see how well the GCMs reproduce the real world. These comparisons can be divided into a few categories that address the questions: Can the GCMs reproduce climate:

- At differing latitudes?
- At different seasons?
- In various regions?

In making these comparisons, the climate community has settled on several quantities that best approximate the system. These are spatial and temporal patterns of precipitation, cloudiness, temperature and OLR. Of course in validating individual processes a much larger number of observed quantities is compared, but these four are most often looked at.

Two of the components of the GCMs — atmosphere and land — are evaluated through intercomparison efforts. The Atmospheric Model Intercomparison Project (AMIP) and the Program for Climate Model Diagnosis and Intercomparison (PCMDI) intercompare some 20 different atmospheric models from around the world[85]. Modelers are asked to present results of runs forced by a standard set of observations. The results are compared against observed quantities such as precipitation, temperatures, etc. Figs. 14 and 15 show some typical comparisons of temperature and precipitation, respectively.

Land models are also intercompared in this way, but there are fewer of them. Ocean and sea ice models are not yet formally intercompared, but a considerable effort goes into validating them against data, and intercomparison projects are beginning.

## Tests Models Should Pass

Ultimately, if the climate models are to be useful for predicting future climates with anthropogenic effects, they must be able to simulate larger integrated observables.

Hartmut Grassl[86] has suggested that the models need to simulate four types of events:

1. The present climate
2. Climate variability since the start of the instrumental record (involving a given set of forcings, and reproducing typical interannual and decadal variability)
3. A different climate from the past such as glacial periods or times of much warmer climate
4. An abrupt climate change event such as the Younger Dryas abrupt cooling during the last transition from glacial to interglacial temperatures

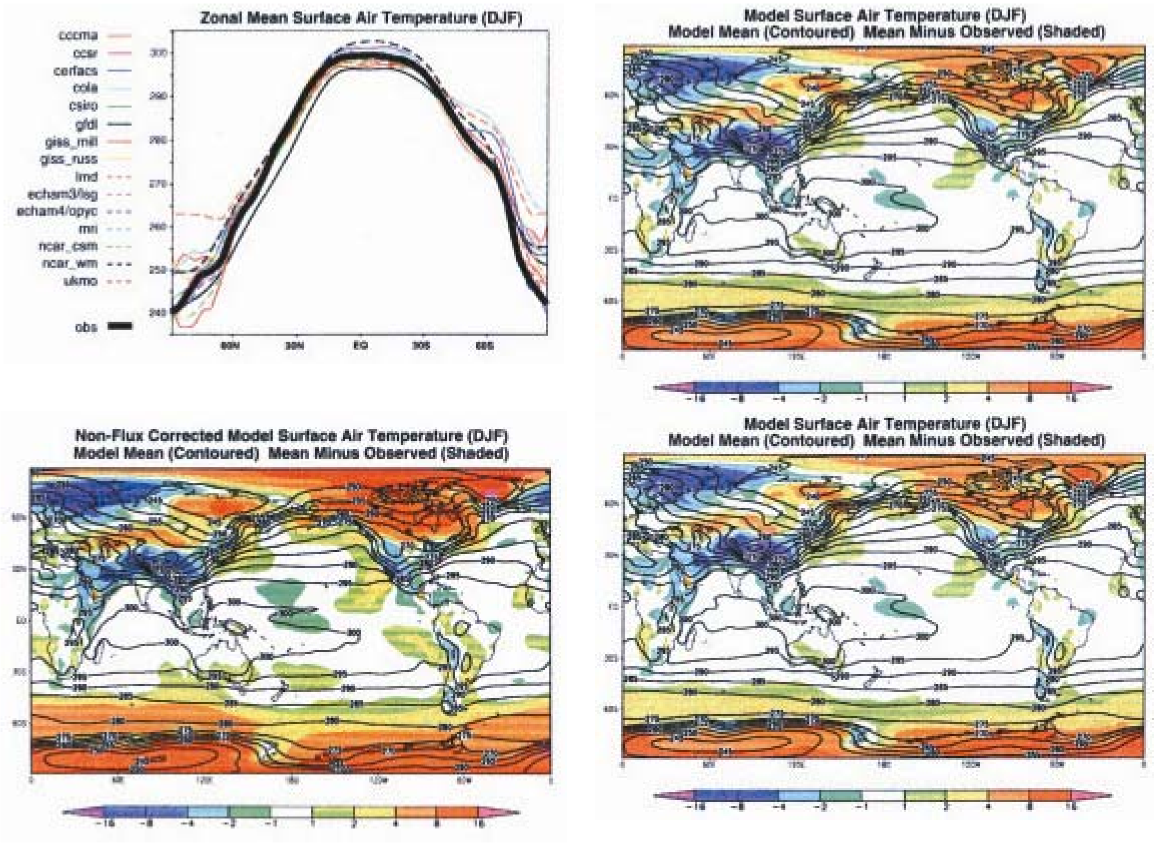

**FIGURE 14.** December-January-February climatological surface air temperature in °K simulated by the CMIP1 model control runs. Averages over all models (upper left), over all flux adjusted models (lower left) and over all non-flux–adjusted models (lower right) are shown together with zonal mean values for the individual models (upper right). Observed value is shown in the zonal mean plot (thick solid line), and the difference between "average model" and observation is shown on the longitude-latitude maps. (From Lambert and Boer 2000)

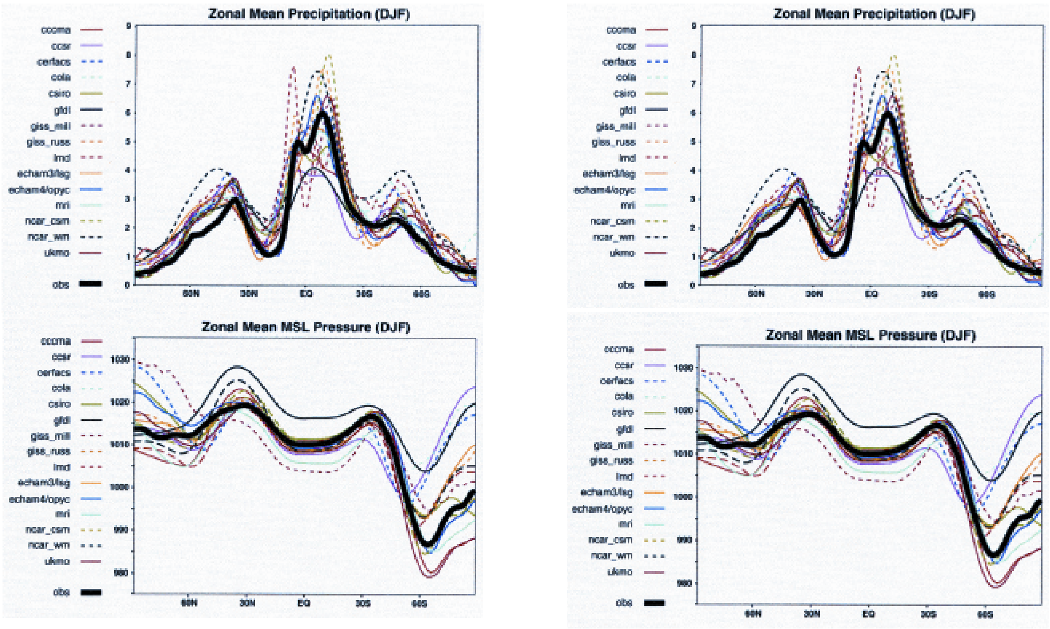

**FIGURE 15.** As in Fig. 14, but precipitation in mm per day (top) and mean sea level pressure in hPa (bottom). Averages over all models are shown at left and zonal mean values for individual models at right. (From Lambert and Boer (2000).)

## The Present Climate

Most GCMs pass this test. First, we recognize that there is a large natural greenhouse effect in operation keeping the average temperature of the Earth above freezing. The average temperature of an airless planetary body at this distance from the Sun is 255°K or –18°C, well below freezing. Water vapor in the Earth's atmosphere is the major GHG and has increased its surface temperature by some 33°C. Computer models simulate this greenhouse effect; however, they often exhibit a slight warm or cool bias, running stably at an average temperature a degree or so different from the observed climate.

Also, GCMs are able to reproduce the seasonal cycle, and they no longer need to be adjusted to keep the ocean from rendering them too cool. Finer vertical resolution in the oceans and better advection methods have largely solved the problem of climate drift. (Some models continue adjust SST and salinity modestly to keep them from gradually cooling.)

## Climate Variability Since ~1850

The natural variability test is more complicated since there are roughly three kinds of natural variability:

1. Those forced by changes in solar activity and by large volcanic eruptions whose aerosols are lofted into the stratosphere where they scatter sunlight away from the Earth
2. Aemiperiodic variations associated with large ocean areas such as ENSO, PDO, NAO, etc.
3. "Intrinsic variability" — chaotic variability of the interactions within the system

The first of these (1) is complicated since there is not general agreement on just how much or in what manner solar variability affects the climate (as discussed above in the previous section).

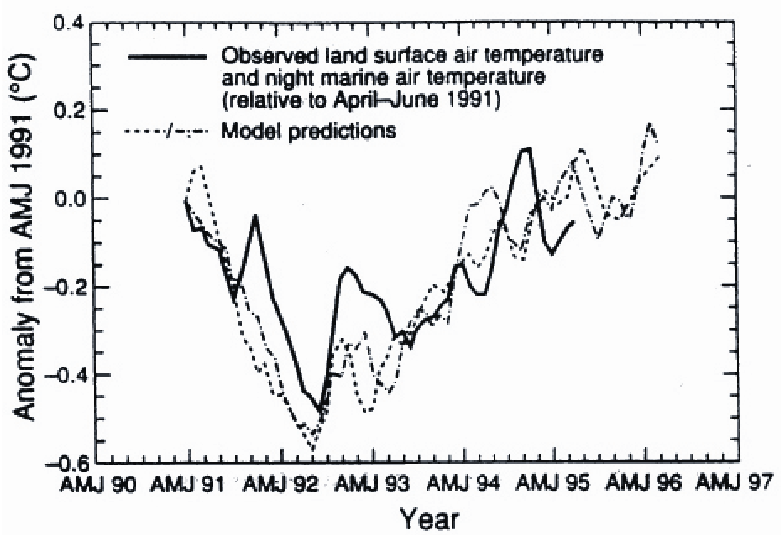

**FIGURE 16.** Predicted and observed changes in global land and ocean surface air temperature after the eruption of Mt. Pinatubo. Lines represent changes of 3-month running mean temperature from April to June 1991 until March to May 1995. The two model lines represent predictions starting from different initial atmospheric conditions.

Since some aspects of solar forcing have yet to be adequately understood and quantified, the ability of GCMs to simulate solar variations must be considered to be adequate but unfinished.

Simulation of cooling due to massive volcanic eruptions, however, has been well done. Following the eruption of Mt. Pinatubo several modelers decided to attempt not only to simulate its effects, but to predict them. Using the rates of decay of volcanic aerosols from the stratosphere based on El Chichon but the estimates of the quantity of aerosols from Pinatubo, they drove their GCMs with the resulting changes in sunlight reaching the lower atmosphere and surface. The codes predicted a rapid global cooling over half a year followed by a more gradual 3- to 4-year warming back to normal climate. The modelers waited to see if their predictions would be correct in both magnitude of temperature change and in timing. The climate performed essentially identically to the models' predictions (Fig. 16). This is a fairly significant result, because it showed that ocean inertia to heating changes was accurately simulated, and, further, it gave a good indication of the magnitude of that inertia in terms of lags between forcing and response (~6 months).

Test (2) is a more stringent test since it requires the GCM's interacting components (in particular the atmosphere and ocean) to produce semiperiodic behavior internally without outside forcing. The most dramatic of these is ENSO, in which El Niños occur every 3 to 7 years. This forcing is indeed chaotic, making precise reproduction of observed ENSO events unreproducible; however, the GCMs are expected to be able to produce ENSO-like events of similar frequency and amplitude. Until recently, GCMs did poorly on this test, but the most recent simulations with higher spatial resolution now seem to be reproducing all the major decadal and interdecadal semi-periodic events — ENSO, PDO, NAO, etc.[87,88,101] and Fig. 17).

On the down side, the models are still not able to predict ENSO events when started from actual climate conditions. They have some skill and do a fair job 6 months ahead of time, but have not been able to extend that to a year or 18 months. Since ENSOs are initiated partly by changes in ocean upwelling, it may be that the model initiation is deficient in capturing deep-water behavior.

Intrinsic variability (3) is of lower amplitude and occurs at all frequencies. It is due primarily to turbulence in the system. Again, the better models are now reproducing fairly well the observed frequency/power curve (Fig. 18).

Test 2 also requires the GCMs to reproduce the observed temperature record over the past 100 to 150 years. To do this, one must drive the climate with both natural and anthropogenic forcings. In addition to reproducing the general rise in 20$^{th}$ century temperatures, the models must deal with several specific problems, the main ones being:

1. Rapid rise in temperature between 1920 and 1945, when AGHGs were at low concentrations; this rapid rise is thought to be due to a marked increase in solar activity, in addition to modest increases in AGHGs (and possibly increases in the North Atlantic thermohaline circulation)
2. Abrupt end to the rapid warming after 1945 followed by actual cooling in the NH until about 1975 with rapid warming thereafter
3. Greater warming at night and during winter
4. Departure of mid-troposphere warming from agreement with continued surface warming after 1992
5. Changes in heating of the deep ocean as observed by Levitus and colleagues[89]

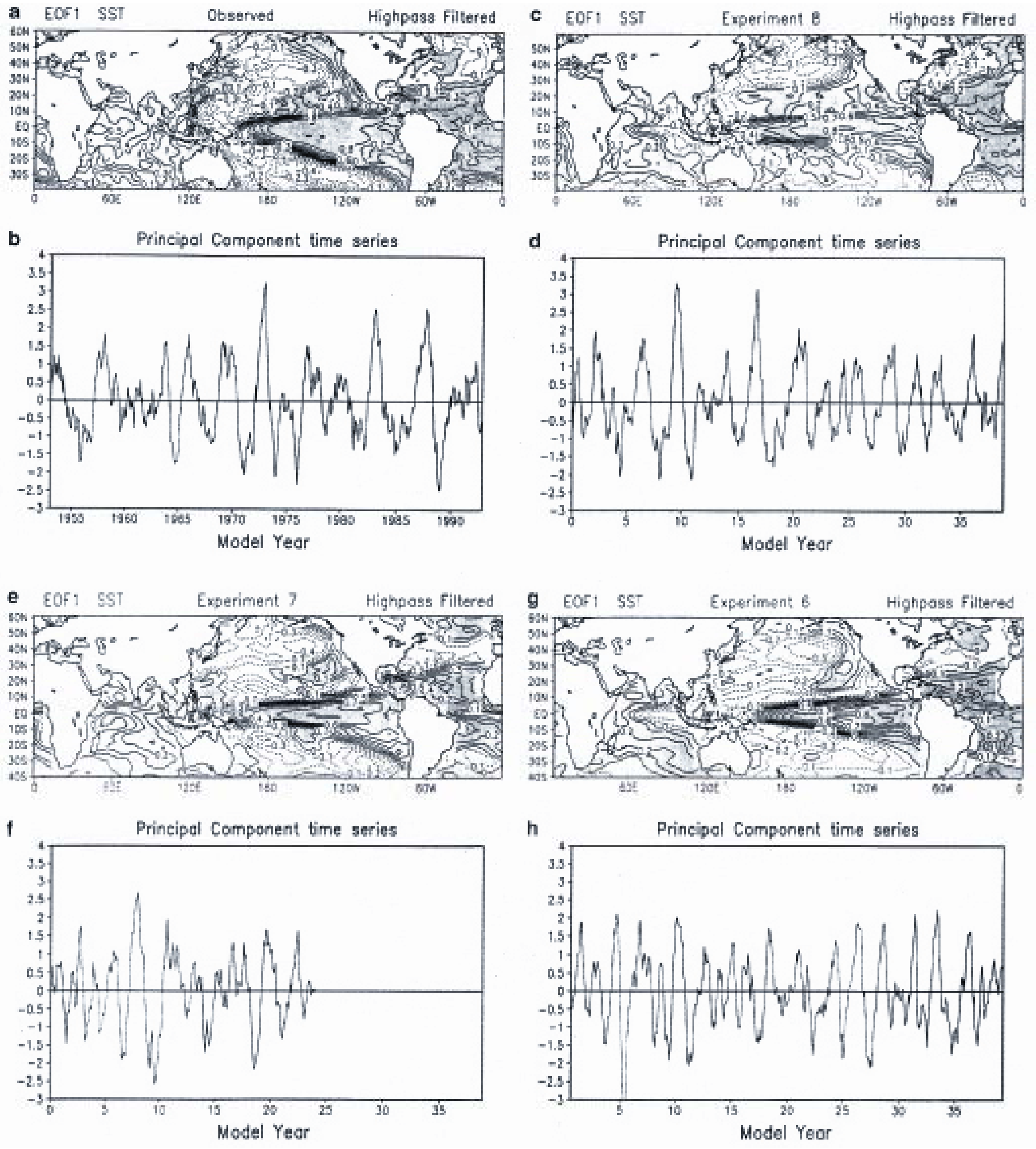

**FIGURE 17.** Comparison of observed El Niño ((a) and (b) from Reynolds and Smith [132] with modeled ((c) and (d) from Washington et al.[101]). a and c are EOF1 from high pass filtered monthly SST, b and d are normalized principal component time series corresponding to EOF1. (From Meehl et al. (2001) Factors that affect the amplitude of El Niño in global coupled climate models, *Clim. Dynamics*, 17, 515–526. With permission.)

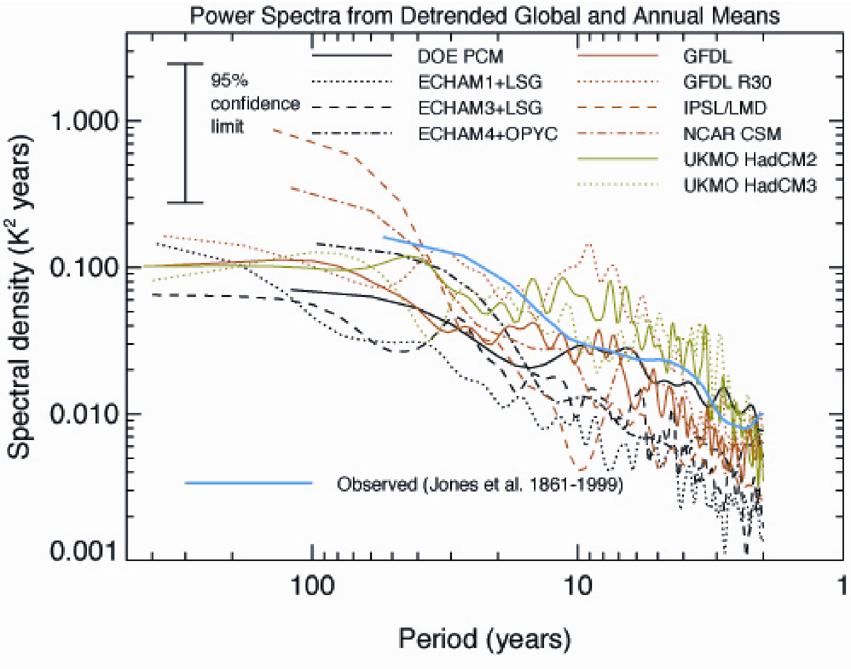

**FIGURE 18.** Power spectra of globally and annually averaged surface air temperature as simulated by the nine longest-running CMIP models. Observed curve was compiled by Jones for the IPCC WG1 SAR.

Many model runs have been made, studying the effects of these forcings. If forced only by increases in AGHGs, all models fail criterion by not warming rapidly enough before 1945, and criterion 2 in that they predict smoothly rising temperatures to levels significantly higher than observed. However, when forcing from increases in solar activity plus aerosols from heavy air pollution following WWII are included, the entire record can be reproduced fairly closely (see "Attribution and What Might We Expect in the Future"). Thus GCMs satisfy criteria 1, 2 and 3. This conclusion has to be tempered, however, in view of the large uncertainties involved in radiative forcing (Fig. 19), particularly that of aerosols and from amplification of warming from feedback.

*Aerosols* alternately scatter, absorb, and reradiate radiation in the atmosphere[90]. Additionally they affect cloudiness and cloud properties. Fig. 19 shows the many different forcings that need to be incorporated into GCMs, few of which include them all. Given the uncertainties of their amount, type and radiative properties, one can see that aerosols might be sufficiently cooling as to completely counter AGHG warming. This review cannot give a detailed discussion of aerosol studies, but will note that new work[91] indicates soot to be more of a warming forcing than previously thought although Ramanathan et al. show it sometimes simply changes the vertical distribution of the heating[92]. The ability of aerosols to either warm or cool the climate complicates any attempt at prediction of future warming, because society may decide to make large reductions in them or they may continue to increase as developing countries advance.

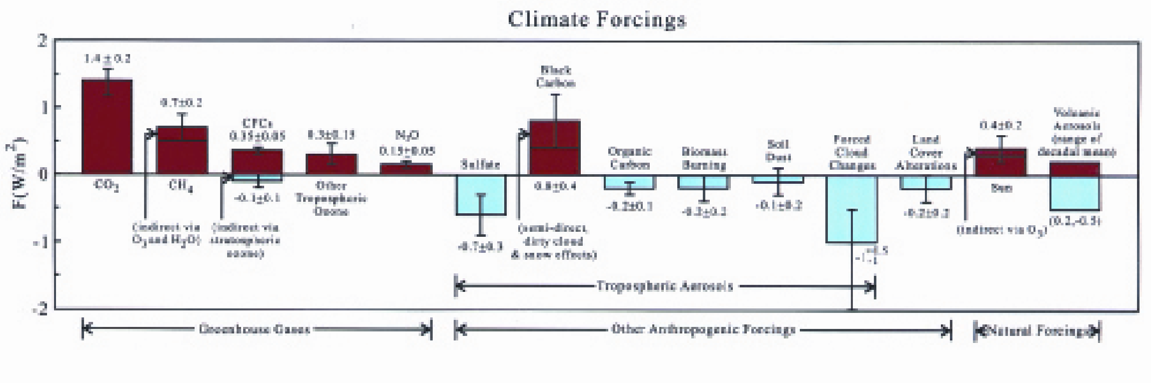

**FIGURE 19.** Estimated change of climate forcings between 1850 and 2000.

*Feedbacks* cause the climate to warm or cool more than it would simply from increases in AGHGs or solar activity. Positive feedbacks are due to increased water vapor, itself a GHG, melting sea ice (changing the Earth's albedo), and possible changes in cloudiness. How much feedbacks dominate is called the model's "climate sensitivity". A standard measure of this is the model's equilibrium response in global temperature rise due to a doubling of $CO_2$ in the atmosphere. Doubling AGHGs produces a temperature increase of about 1.3°C. But feedback amplification increases this warming to between 2.0° and 4.0°C depending on the model used (see Fig. 22 later). This is very important for studies of greenhouse warming since high sensitivity models predict larger temperature rises in the 21$^{st}$ century than the lower sensitivity ones. And so it is important to find ways to determine which sensitivity is correct. This involves validating the feedbacks in the model, which is very difficult to do. Studies of paleoclimates both considerably warmer and cooler than present give a similar range in feedback uncertainty, lending credence to the models but not reducing the uncertainty[93], and so considerable work is being done to improve treatment of water vapor and clouds in the models.

Water vapor feedback is especially difficult to determine because it depends on the vertical distribution water vapor[94]. In the boundary layer close to the Earth's surface, evaporation is a fairly well-known function of temperature and wind speed. On the other hand, in the free troposphere above, where vertical convection interacts with the large scale flow, our understanding is considerably less (models tend to be too moist high in the troposphere). This is disquieting since it is here that increases in water vapor produce the large responses to increased greenhouse gases. Improvements are complicated because observations are either lacking or inadequate.

Cloud feedback is closely related to water vapor, which complicates uncertainties in formation, type, altitude, duration, extent and perhaps most of all interaction with radiation. Clouds mostly scatter visible, shortwave radiation (SWR) while they absorb and re-radiate infrared, longwave radiation (LWR). However recent observations suggest that they absorb about 20% more SWR than previously thought, which may be due to soot particles in the droplets. The behavior of both SWR and LWR in clouds depends on many factors: thickness, droplet size, total moisture content, aerosol content and effects. High clouds warm by re-radiating LWR downward, and low clouds cool by upward reflection of SWR in addition to radiating LWR. Satellite observations show that net radiative cloud forcing is negative[95], causing cooling of the climate system. However, their feedback response to warming is poorly known — even to the sign of the forcing! In addition clouds produce precipitation. Not only is this a key quantity important to humans, the attendant phase changes during the precipitation and re-evaporation process are important to the total heat transfer within the atmosphere as well as to the vertical distribution of moisture. Thus, studies of the microphysical processes involved are an important part of climate research. In addition they are necessarily subgrid scale in size and so must be parameterized.

### Departure of Mid-Troposphere Warming from the Surface after 1992

Until very recently all models failed criterion 4 in that they predicted warming aloft would be as much or slightly greater than at the surface. This prediction was largely true prior to 1992, but, following the cooling caused by Mt. Pinatubo's eruption, warming in the mid- and upper-troposphere slowed markedly nearly to zero while observed surface temperatures continued to rise. Models are only now beginning to explain how this is happening. (For a discussion of this conundrum, see "Surface vs. Satellite Temperatures".) Many think the problem is inadequate treatment of energy exchange between the stratosphere and troposphere. For most models vertical resolution is too coarse and/or ozone depletion is not precisely included, and thus they do not adequately treat the observed dramatic cooling in the stratosphere since 1992. The stratosphere is currently cooler than any time since observations began 50 years ago. This could cause the upper troposphere also to be cooler, thus changing the temperature lapse rate so as to reduce warming aloft below what it is at the surface. Spatially one-dimensional models show this effect[96].

It remains for complete GCMs to attempt this difficult calculation comprehensively. It has been attempted in at least two recent simulations, those done at the Max-Plank-Institute (MPI)[97], and more recently at NASA's Goddard Institute for Space Studies[18]. The MPI runs did not include solar variability but made a rather detailed study of the sensitivity of the model to successive inclusion of GHGs and various amounts of aerosols. They reproduced surface temperatures rather well, particularly in the second half of the $20^{th}$ century. These runs were significant in that they included reduction of sunlight by Pinatubo's stratospheric aerosols and attendant reductions in ozone. This resulted in initial stratospheric warming (about 1 year) due to absorption of sunlight by the aerosols. But as these settled out, the stratosphere cooled due to ozone loss, matching observations. Apparently this cooling affected temperatures in the troposphere below. Thus, subsequent warming in the upper and middle troposphere did not keep pace with the surface. The MPI simulations approximated this effect, but only until 1995. After that the model predicted more warming than has been observed. Nevertheless, inclusion of a rough approximation of stratospheric cooling seemed to be pointing in the right direction.

The NASA team did a much more detailed study, including sunlight reductions from five volcanoes (Agung [1962], El Chichon [1982], and Pinatubo [1991] and two smaller ones) and more accurate stratospheric ozone reduction. The resulting temperature results compared well with the observations at all levels (Figs. 20a, b, and c). Fig. 20d shows that these forcings do not entirely translate into warming, because there is a growing imbalance in planetary energy while the oceans take up some of the heat. Finally the run reproduced rather well the observed decline in ocean ice cover in the last 50 years (Fig. 20e). But the code still predicted more warming in the troposphere than the satellite observed after 1995. Further runs suggest why. While this simulation used an improved atmospheric model, it was coupled to a very simple two-layer ocean model. When the runs were redone with a full ocean model (the hybrid coordinate ocean model[98]) the satellite MSU2LT record was more closely reproduced even after 1995[99]. These results need to be studied more closely, but if they hold up, this is the first time criterion 4 has been satisfied.

Two points are worthy of mention. First, the result seems to come from two improvements, one at the bottom of the troposphere and one at the top. Second, the ocean result was completely unforeseen because the ocean researcher doing the study was not aware of the atmospheric problem and was concentrating only on the difference in ocean behavior between the simple and the complex ocean codes. Thus no "tuning" was done to achieve this result.

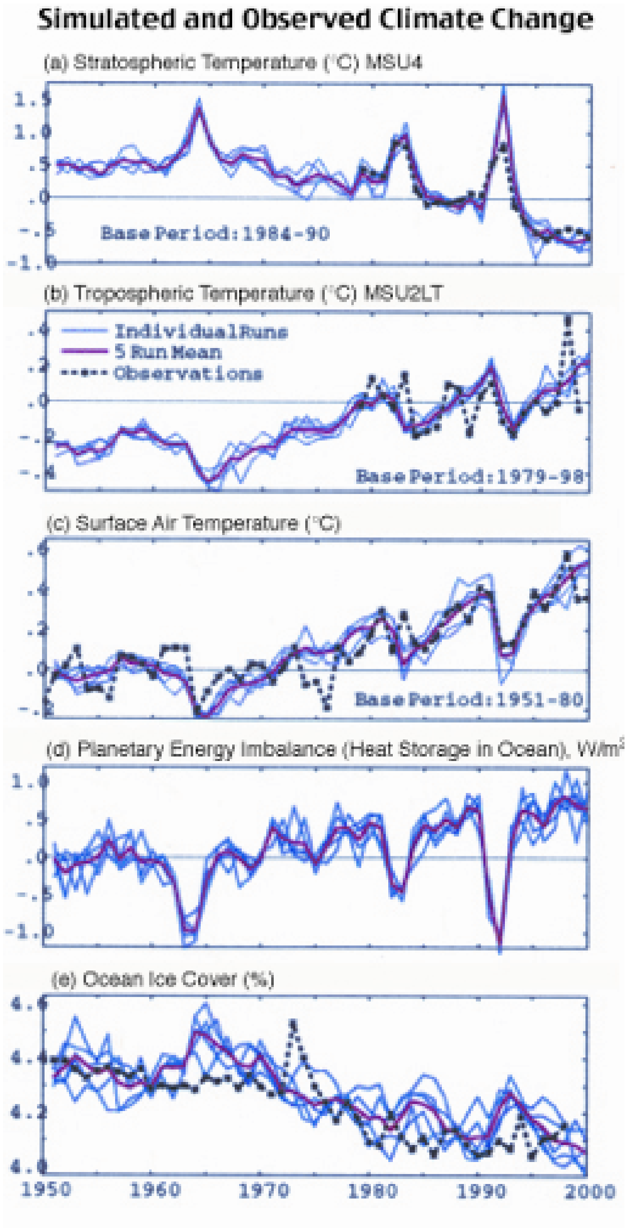

**FIGURE 20.** Simulated and observed climate change for 1950–2000. Note in (b) failure of model to reproduce observations after 1995.

## COUPLING BETWEEN OCEAN AND ATMOSPHERE

Test (5) is also a particularly demanding one since it asks how well the coupling between ocean and atmosphere is modeled, i.e., does the code accurately handle fluxes of temperature and water vapor between ocean and atmosphere. At least three teams have simulated the observed deep ocean temperatures, but with an important twist. Attempts by modelers to reproduce 20$^{th}$ century temperature records have always been done with the answer known beforehand. Thus, critics could charge that the modelers simply “fudged” parameterization constants to get the right answer. This is true to some extent. For example, codes with low climate sensitivity need to add less aerosol cooling to pass test (2) (climate cooling 1945–1975) than do those with high sensitivity. But this is not how at least one of the ocean heating studies was done. It found good agreement with ocean observations in a set of climate runs done *before* the ocean data were published. Following publication of the ocean data, Barnett et al.[100] analyzed ocean information from simulations already done by NCAR's Parallel Climate Model (PCM) group. (The PCM derives its name from its formulation for large parallel processing computers[101].) The PCM group had been attempting to reproduce the 20$^{th}$ century temperature record, and had done a reasonably good job of it. But little attention was paid to resulting deep-ocean heating. Barnett's group, however, concentrated on the oceans. Fig. 21a shows comparisons with Levitus data ocean by ocean, and Fig. 21b compares model and data over the depth ranges indicated. The conclusion is that, while the model does not reproduce some of the decadal variability, it does do rather well in simulating heat uptake for all the oceans at various depths. This is not so much a message that the models are doing really well. It is more in line with what Richard Kerr wrote in an editorial accompanying these two papers. He termed it "another test passed"[102]. At least two other modeling groups have reproduced this data[18,103].

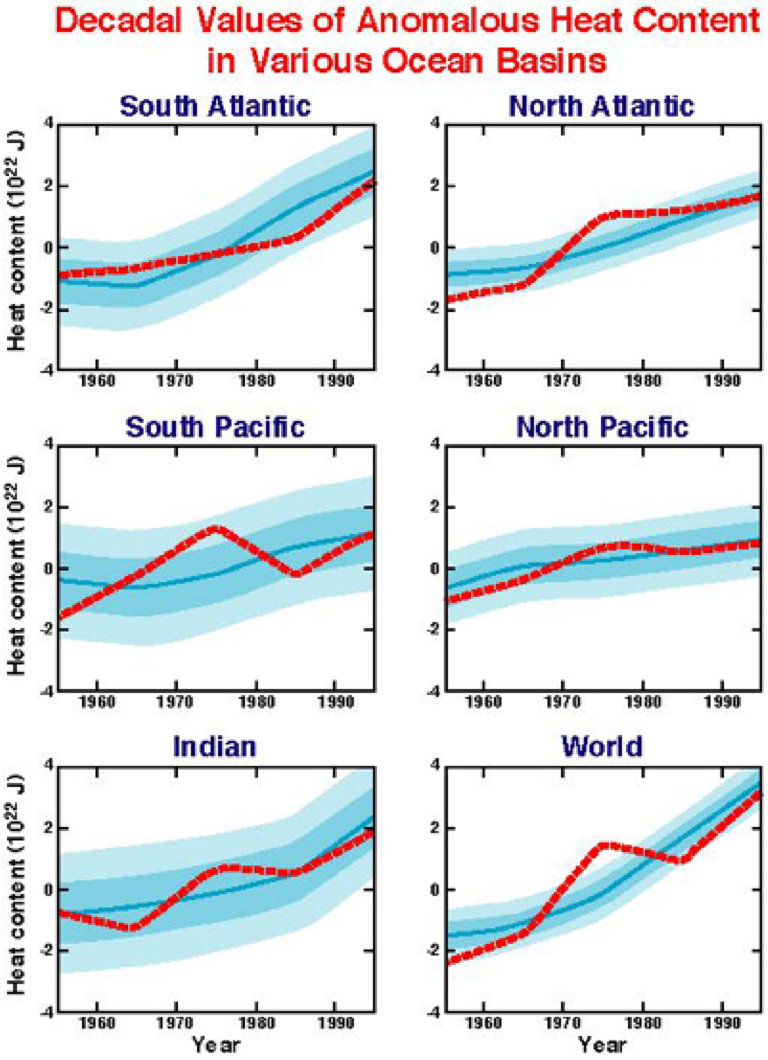

**FIGURE 21.** Comparisons of observations and modeled ocean heat content/temperatures. (a) Decadal values of anomalous heat content in various ocean basins. The heavy dashed line is from observations and the solid line is the average of five realizations of the PCM forced by observed and estimated anthropogenic sources. The shaded bands denote one and two standard deviations about the model mean. (b) Decadal temperature anomalies (°C) in various ocean basins since 1870 from the PCM. Gray-shaded regions indicate signals statistically distinguishable from zero.

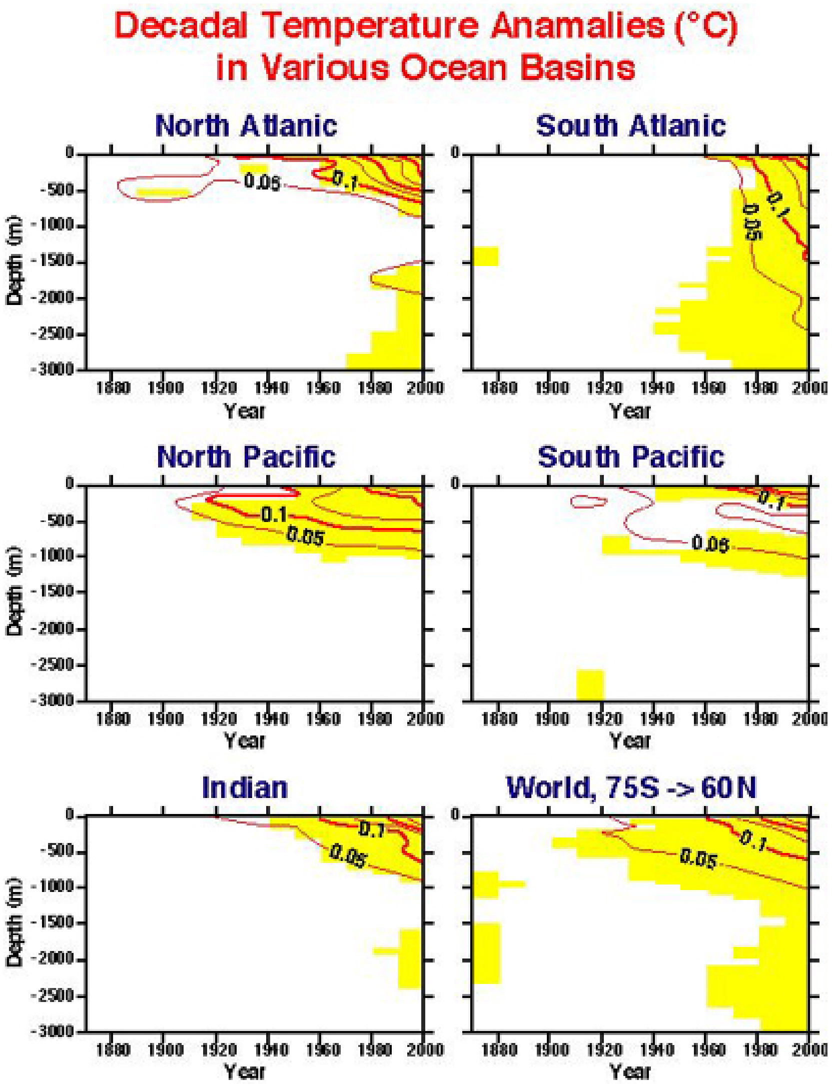

FIGURE 21B

## Paleoclimates and Transitions

Tests (3) and (4) are passed more or less well, but are hampered by large uncertainties in paleo-forcings. Moreover, less work has gone into these studies. Space does not allow detailed consideration of this effort but, in summary, it is clear that some previous climates cannot be reproduced if the data are correct. Rapid climate jumps are only simulated when some significant outside forcing is introduced or when changes occur in the North Atlantic thermohaline circulation. Much more work needs to be done in these areas.

## Summary

GCMs are actually a coupled combination of four models: atmosphere, ocean, land, and sea ice. Comparisons with observations show them to reproduce reasonably well large-scale elements of climate and both seasonal and latitudinal variations. They also reproduce natural variability and responses to anthropogenic forcings, thus simulating the 20$^{th}$ century temperature record. They have shown skill in predicting such features as climate response to large volcanic eruptions and deep sea response to atmospheric warming. However, necessarily coarse spatial resolution renders them unable to reliably simulate regional climate, and large uncertainties in aerosols, clouds, and water vapor feedbacks translate to similar uncertainties in their ability to simulate departures from present climate.

## ATTRIBUTION AND WHAT MIGHT WE EXPECT IN FUTURE (*YOU CAN'T PREDICT THE FUTURE*)

Encouraged by the successes of GCMs but cautioned by their obvious deficiencies, these essentially research climate models have been used to explore what climate changes we might expect if AGHGs continue to increase at present rates. Since the 20th century experienced significant increases in these gases, it provides an observational basis to test the ability of these models to react to increasing forcings. The approach used has been to add these forcings incrementally as successively accurate approximations of the real world are desired. As discussed in the previous section, simulation of this complex record requires incorporation not only of AGHGs but of sulfate aerosol and solar forcings. Continued study has resulted in inclusion of a rich and complex soup of minor forcings, which, however, taken together are significant.

Though GCMs have been increasingly successful in reproducing the past-present interface, it became immediately clear upon predictions of the future that they differed in climate sensitivity due to differences in how they simulated feedbacks (see previous section). Predictions of the amount of warming/change for a given scenario of increased AGHG in the 21st century are a function of how strong these feedbacks are (Fig. 22).

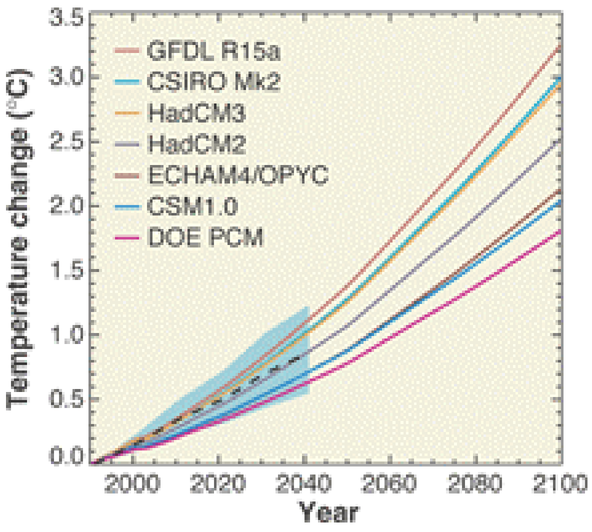

**FIGURE 22.** Predictions of warming in the 21st century by six models. Note these differ primarily due to their differing climate feedback "sensitivity". (From Allen et al. (2001) *Science*, **293**, 430. With permission.)

## Five Recent Simulations Employing Multiple Forcings

As examples of how well different GCMs are able to predict future warming, we will discuss several representative ones, noting their spatial resolution, forcings and other model approximations, first showing how well they reproduce the 20$^{th}$ century and then giving their predictions. Note that due to the essentially chaotic nature of weather and climate, most studies "run" the climate several times from different starting points in a control run. The resulting ensemble gives estimates both of the model's internal variability and an average prediction. Thus, there is no exact future climate, only a range of responses to minute differences in starting conditions. In actuality our climate will be only one such realization, but would be expected to lie within the envelope of the ensemble realizations.

### *The Geophysical Fluid Dynamics Laboratory (GFDL) Model*

This example is given not so much to show prediction of the future, but to show that the spread in internal variability from different realizations can be large enough to give the observations of early 20$^{th}$ century climate without the accepted necessary recourse to solar forcing. The GFDL model was used to study climate from 1900 to 2000 with emphasis on the early 20$^{th}$ century[105,106]. This is similar to GFDL's earlier GCM, but with higher resolution: atmosphere 3.75° longitude by 2.25° latitude with 14 vertical levels, ocean 1.9° longitude by 2.25° latitude with 18 vertical levels. Ocean/atmosphere flux adjustments are used. A thermodynamic sea-ice model is used over oceanic regions with ice movement determined by ocean currents. Five runs were started from different points in a 1000-year control. These included observed AGHGs and aerosol concentrations and forcings (aerosol radiative perturbations approximated as albedo changes). The models included no forcings from solar variability nor volcanic eruptions. Yet one (Experiment 3) of the runs matched observations very well over the entire 140 year period! Not only did it match the global temperature record, but it matched fairly well the zonal (meridian averaged) behavior, as shown in Fig. 23. In addition it matched the main spatial character of the observed warming (Fig. 24).

These figures show that the observed warming was concentrated in the North Atlantic region and northern parts of North America. Similar results were obtained with Experiment 3. The authors note that this is due to enhanced thermohaline circulation (THC) in Experiment 3 and that, "The enhanced THC appears to play a role in the warming through an increased meridional transport of heat and an increased ocean-to-atmosphere heat flux." They also note that this enhancement "is at least partially attributable to a persistent positive phase of the model-simulated NAO."

This is worth keeping in mind as it points to the ability of NAO to create hemispheric warming, and it shows (Fig. 23) that warming prior to 1950 was not nearly as globally uniform as after 1970. This, plus the fact that, even without solar activity forcing, this model (as well as other models discussed below) is able to "catch up" with the observations after 1970, points to AGHGs as becoming dominant in a way that differs from ocean circulation warming and perhaps from other forms of natural forcing.

### *United Kingdom's Hadley Center model, HadCM3*

This model, run by the United Kingdom's Hadley Center, included both natural (solar and volcano) forcings as well as those from anthropogenic sources. An ensemble of four separate simulations was done[107]. Initial conditions were taken from hundred year intervals of a 1300-year control run. Forcings included individual GHGs ($CO_2$, $CH_4$, etc.) stratospheric and tropospheric

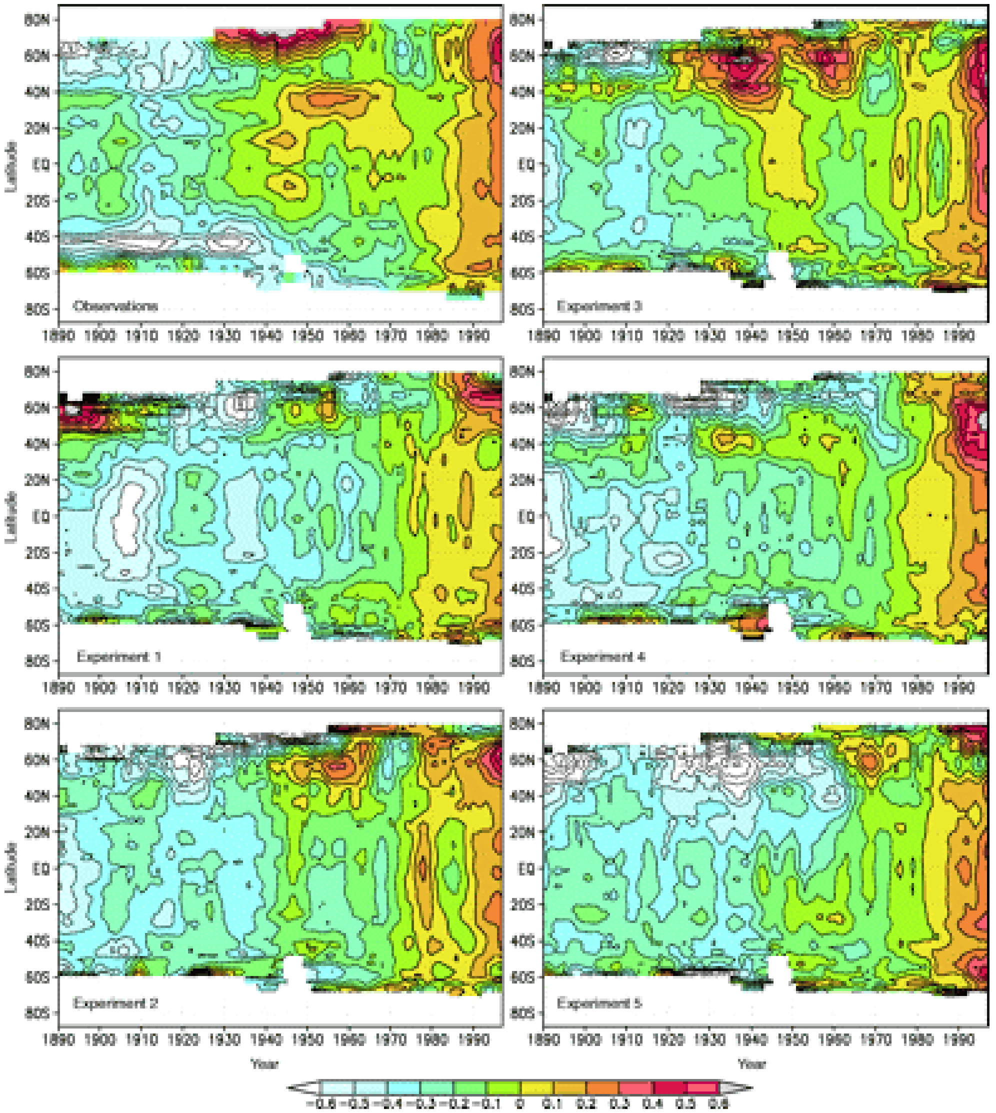

**FIGURE 23.** Zonal mean anomalies of surface temperature (in °K) for observations and the five numerical experiments as labeled. Note the experiment agrees fairly well with the observations.

ozone, sulfur dioxide with the emission, transport, oxidation and removal of sulfur species treated. Indirect aerosol forcing was also represented. Natural forcings were stratospheric aerosols following volcanic eruptions and spectrally resolved solar irradiance changes. This ensemble was then compared with two others, one with only natural forcing and one with only anthropogenic. These were called ALL, ANTHRO and NATURAL. Fig. 25 shows the results of these simulations. NATURAL reproduces the first half of the 20$^{th}$ century but not the second, largely because of multi-year cooling from three volcanoes, as well as lack of AGHG forcing. ANTHRO misses the first half but gets the second. The two together in ALL succeed in reproducing the entire record fairly well. The ALL simulation is then extended through the 21$^{st}$ century driven by the IPCC's Special Report on Emissions Scenarios, B2, which assumes a more modest increase in GHGs than previously assumed. Stratospheric ozone is assumed to recover as stratospheric chlorine loading diminishes, and natural forcings are assumed to remain at 1999 levels (high solar, no volcanoes). The HadCM3 model has significant "climate sensitivity" and predicts a 2.7°C temperature rise between 1945 and 2100.

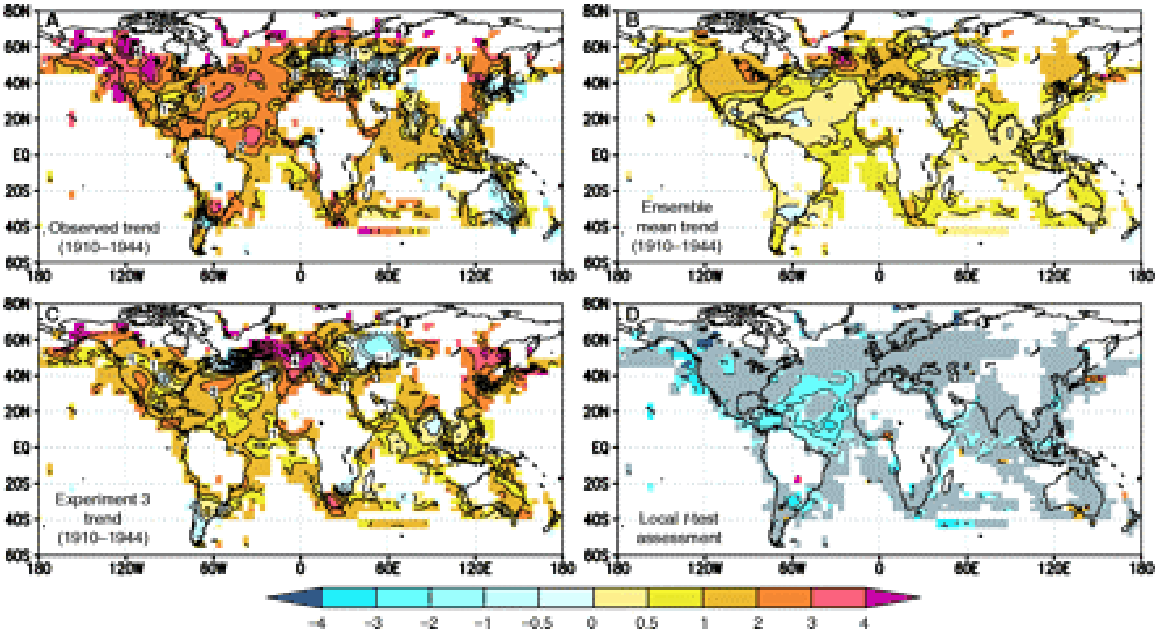

**FIGURE 24.** Linear trends of surface temperatures (°K/100 years) as labeled. Note agreement between observations and Experiment 3.

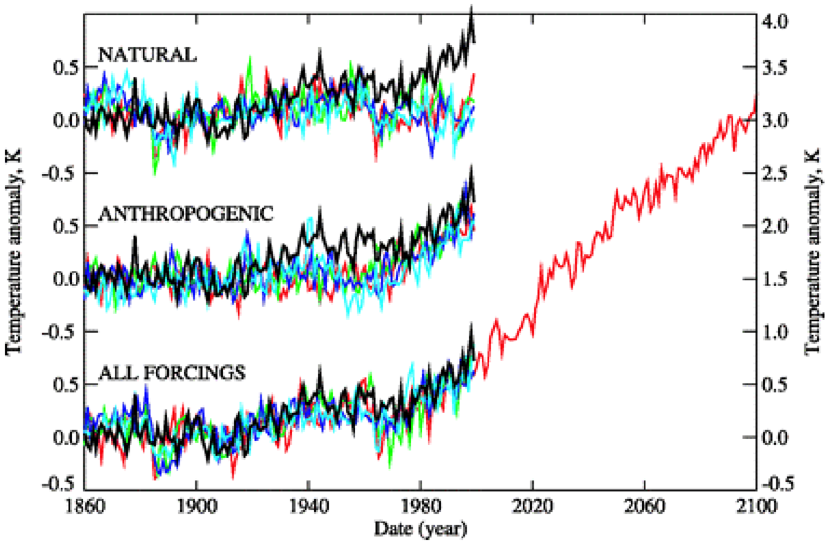

**FIGURE 25.** Annual-mean global surface temperature anomalies (relative to 1881–1920). Black curves are the observations, colored are individual simulations (see text).

This model compares well, not only with globally averaged observations, but also with those averaged over land and sea, NH and SH, as can be seen in Fig. 26, reproducing the observed larger warming over land than sea particularly in the NH.

The largest discrepancy is that the model underestimates the rapid warming in NH oceans. This might be expected since the North Atlantic Ocean was observed to warm rapidly very likely due in part to some chaotic internal variability such as a change in the THC that the model does not capture at that time.

Finally, we can inspect the ability of the model to reproduce the general warming trends across the global as well as get an appreciation for the level of observational coverage being used for comparison in Fig. 27. Here we find plotted for three time intervals (1910–1939, 1940–1969 and 1970–1999) observations, simulations and their difference across the globe.

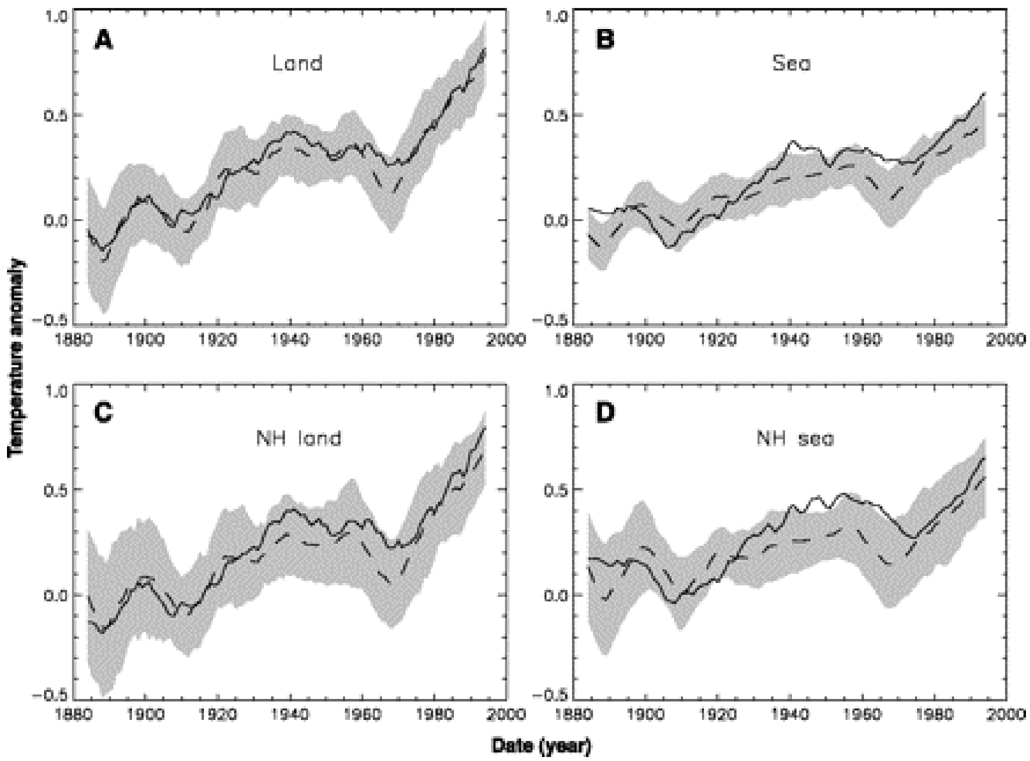

**FIGURE 26.** Decadal-averaged, surface temperature anomalies spatially averaged as labeled.

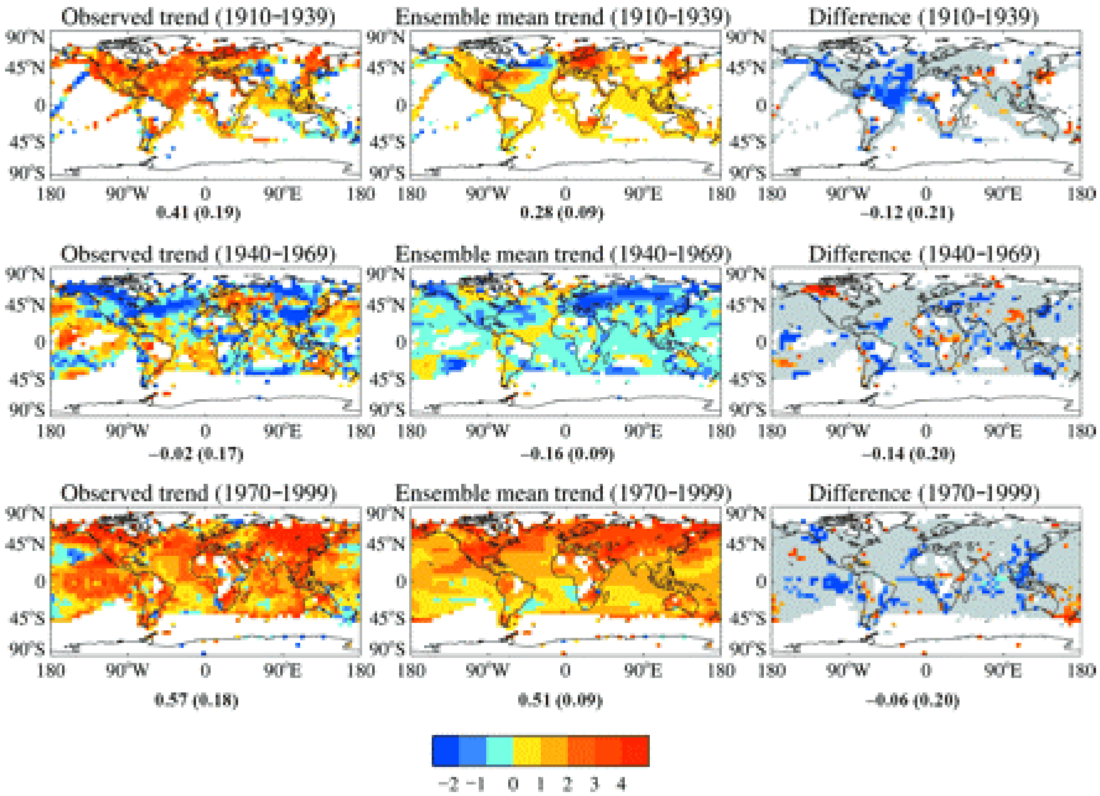

**FIGURE 27.** Linear trends (°K/30 years) of near-surface temperature for three time intervals (1910–1939, 1940–1969 and 1970–1999) observations, simulations and their difference across the globe.

During the cooling period, 1940 to 1969, the marked hemispheric difference in the observations is not reproduced very well in the simulations. Since the cooling here is caused largely by industrial air pollution (fairly clear from the model results), one might question whether some other source of NH cooling was at play or whether aerosol cooling might not have expressed itself in a different spatial distribution. Regardless the SH cooling in the model seems strange.

Finally the authors caution, "Given the uncertainties in historical forcing, climate sensitivity, and the rate of heat uptake by the ocean, the good agreement between model simulation and observations could be due in part to a cancellation of errors — for example, too high a climate sensitivity combined with too low an historical forcing. Hence our result does not remove the need to reduce uncertainty in these factors." In this regard, it would be interesting to have this model analyzed to see how well it reproduces the Levitus deep ocean warming data (see discussion under "Computer Simulations").

Along these lines the authors point out that the model "simulates roughly half the present-day concentrations of sulfate aerosol observed over Europe and will therefore underestimate their cooling effect. However, there may be some compensation from omitting the warming effect of black carbon aerosols."

## *National Center for Atmospheric Research (NCAR) models, CSM and PCM*

CSM 1.3 — The Climate System Model[108] — is a fairly new GCM without need for flux adjustments. The reported runs were forced only by AGHGs and direct sulfate aerosols. Forcing scenarios for the 21st century are BAU (Business as Usual to 710 ppmv $CO_2$ by 2100) and a STA550 (Stabilizing to 550 ppmv $CO_2$ by 2001). CSM1.3 has low climate sensitivity of 2.1°C for doubling of atmospheric $CO_2$. In CSM1.3, the atmospheric spatial resolution is fairly good (~2.8° horizontal resolution and 18 vertical layers with the top layer at 4.8 mb, high in the stratosphere). The oceanic component of CSM1.3 has 2.4° resolution down to 1.2° at the equator and 45 vertical levels. Thus, excepting for limited case studies, this model is among the most highly spatially resolved yet run. 20th century runs were driven by observed GHGs and aerosols with no solar or volcanic forcing. After 1990 stratosphere ozone was computed by an outside chemistry model. GHG concentrations are computed in complicated manner.

The two runs (using different estimates of increases in AGHGs) predict warming between 1950 and 2100 of 1.7°C (STA550) and 2.1°C (BAU). Thus this prediction is definitely low end, less than 1.5°C more than at present! But this warming is the average of land and ocean, which differ — 1.0 to 2.0°C for ocean and 1.5° to 3.0°C for land. Fig. 28 shows predictions of global temperature rise in the 20th century.

A variation of CSM, the Parallel Climate Model (PCM), employs more sophisticated and high resolution ocean and sea ice models[102] (Fig. 17). It has similar low climate sensitivity.

## *Max-Plank-Institut für Meteorologie Model*

The Max-Plank-Institut für Meteorologie, Hamburg, Germany has been doing attribution calculations for years. Recently a landmark simulation was completed that included volcanic and ozone forcings in the stratosphere in a more realistic manner than before[97]. An additional study of climate dependencies on various approximations to sulfate forcings was done. The GCM employed atmospheric and ocean horizontal resolution of 250 to 300 km[109]. Air/sea fluxes of momentum are unconstrained, while fluxes of heat and water vapor are flux adjusted, but only as annual averages. The model simulated the ENSO phenomenon quite realistically. Using the IPCC's IS92a scenario for changes in GHGs etc., a study of climate was done from 1863–2050

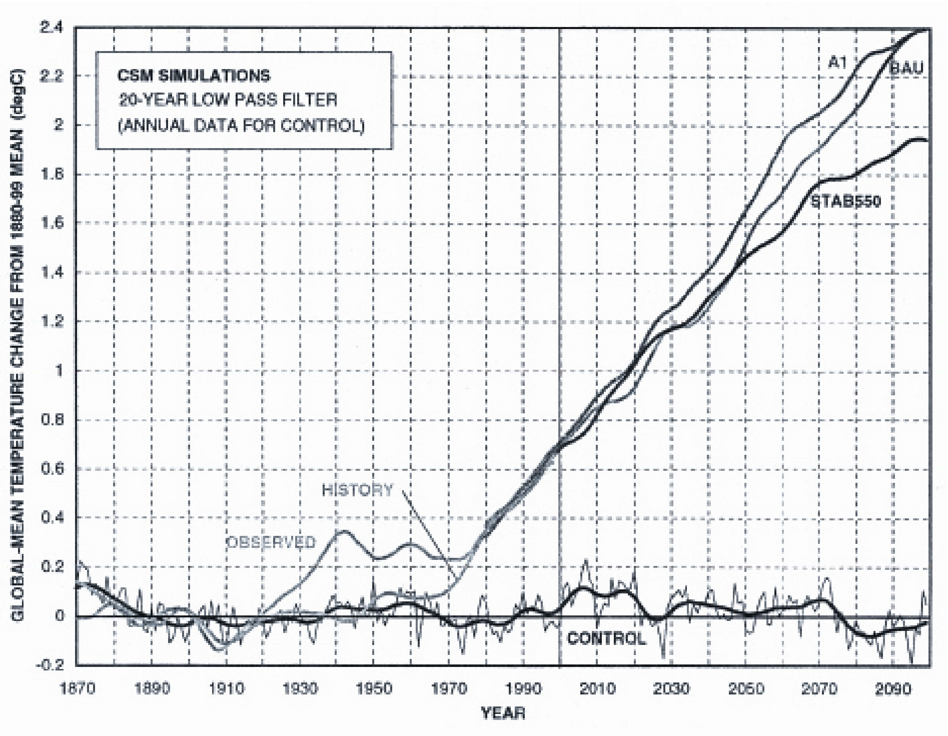

**FIGURE 28.** Global temperature predicted by the model for two scenarios, BAU and 550.

with emphasis on the present climate. Following a long control run in which forcings remained constant, several experiments incrementally added forcings from GHG only, through aerosols (direct and indirect). As expected, the GHG only experiment overpredicted warming, but the full forcing run simulated observations rather well except for missing the warming in the first half of the 20$^{th}$ century (lack of solar forcing). Using this run as a control, the effects of the Mount Pinatubo eruption and subsequent changes in stratospheric ozone were simulated. This study was discussed in the section on computer simulation and resulted in the first simulation of mid-troposphere temperature anomaly differences from those at the surface (Fig. 29). Following 1995 the model predicted more warming than observed in the mid-troposphere, and less cooling than observed in the lower stratosphere. (One wonders if the stratospheric cooling had been better simulated, would the mid-troposphere data have been reproduced after 1995.)

## NASA Goddard Institute for Space Science (GISS) Model

The final modeling exercise is also the most recent[18] (submitted for review and so results must be taken cautiously). This study, presented at a Senate hearing (May 2001) used the new GISS SI2000 atmospheric GCM[110]. Although it has fairly coarse spatial resolution (horizontal is 4° × 5° with 12 vertical layers), it concentrated on using the most comprehensive set of forcings to date. Fig. 20 shows these forcings. Of particular interest is inclusion of forcing due to soot, which was estimated at a whopping 1 W m$^{-2}$. Also included are natural forcings: solar and that from five volcanoes since 1963. Finally included are changes in stratospheric chemical constituents. A potential weakness is the use of fairly primitive ocean models. The results have already been shown above (see “Computer Simulations” and Fig. 20). That discussion includes additional unpublished results using a better ocean model that show even better agreement with observations, leading to increased confidence that models can indeed reproduce this nonintuitive vertical temperature structure.

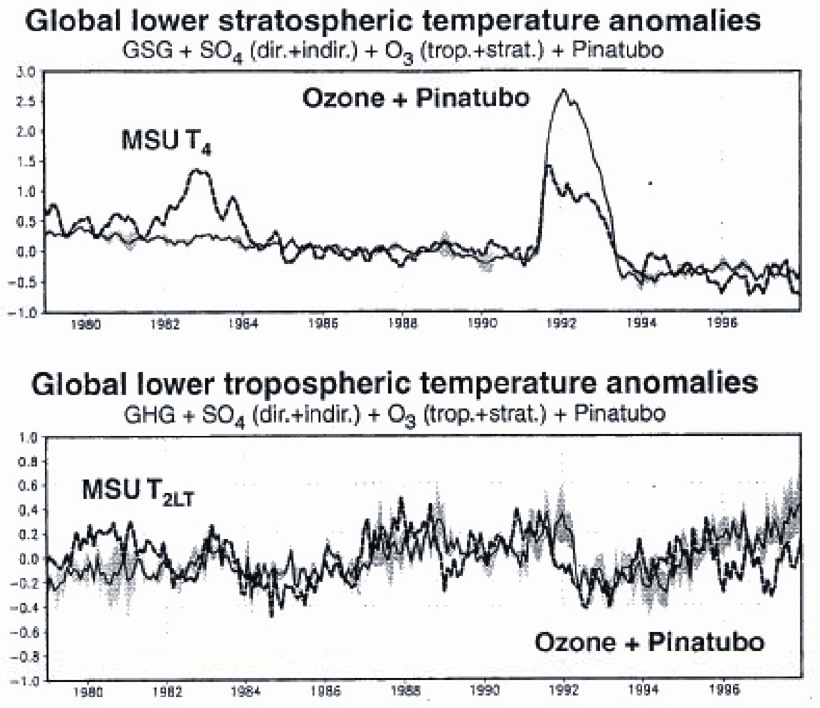

**FIGURE 29.** Comparisons of model results with satellite observations for lower stratosphere (top) and mid-troposphere (bottom). Dashed lines are observations, solid model are results and shading indicates range of the ensemble runs. Note agreement between MSUT2LT after Pinatubo (1991 till 1995).

Having simulated the global climate, the study goes on to make two predictions for temperature change through 2050. For a BAU scenario, the rise from 2000 to 2050 is 1.5°C, while for a suggested scenario (featuring significant reductions in non-$CO_2$ GHGs and reprising the five volcanic eruptions that occurred 1963–1991) from 2013 to 2041, the rise is 0.7°C (Fig. 30). To put this into context, the results are compared with similar simulations done in 1988 that predicted temperature changes for three scenarios. The actual warming thus far is following the 1988 "slow growth" path, but the proposed scenario would cause even less warming. This comparison with 1998 results shows another important aspect of climate modeling and scenario building, namely that it has not changed much in more than 10 years despite considerable improvements in the GCMs and in the scenarios.

## More Sophisticated Attribution Methods

Merely reproducing such gross observables as large-scale temperature averages is not sufficient for selecting anthropogenic global warming over other possible causes of climate change. It is recognized that different sources of warming might each have unique effects on aspects of the climate. For example, GHG forcing is expected to cause warming preferentially at night and during the winter, while one might expect increased solar activity to cause warming when the sun is shining. Aerosol cooling might be expected to cool the NH more than the SH since most industrial and transportation pollution is concentrated in the north. There have been several attempts to find these so-called "fingerprints" of GHG forcing on climate[111]. However, because the ocean/atmosphere system moves heat around in chaotic ways, such fingerprints are less obvious than originally expected. For example, GHG forcing should have an increasing effect going from equator (where water vapor can swamp the small additional GHG forcing) to poles (where, in the relative absence of water vapor, anthropogenic GHGs should dominate).

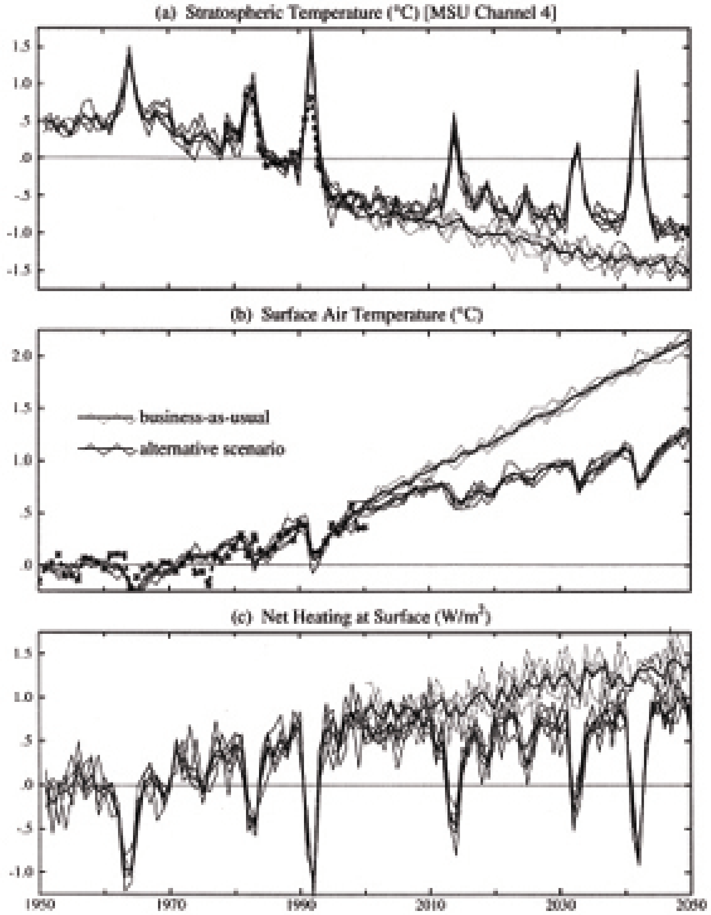

**FIGURE 30.** Stratospheric temperature, surface temperature, and heat flux into the Earth's surface for SI2999 model with six forcings for two scenarios: "Business as Usual" and proposed "alternative".

Thus, one might expect (and most models predict) that warming at high latitudes will be larger than at low latitudes. This is observed in the NH over land up to quite high latitudes, but in the polar regions themselves there has been little warming. And so, if global warming is largely due to humans, other negative forcings must be affecting polar regions.

Attempts to quantify attribution have been published[112,113]. These methods are fairly complicated and, due to space limitations, will only be referenced here. Suffice it so say that they too show the AGHG theory to account for the observations (although other theories have not been subjected to such a test).

## Alternative Ways to Reproduce the 20$^{th}$ Century Temperature Record

What other forcings could account equally well for the temperature record? Two are currently under discussion: solar indirect (more than just due to changes in radiation) and soot (aerosols that absorb sunlight rather than reflect it[91]). Both have been claimed to be large enough to account for most of the warming, and a clever combination in which the sun does most of the first half century and soot assists in the second half is attractive to some.

How does one choose between these forcings? First one looks at the physics:

1. $CO_2$ forcing is well understood and, because $CO_2$ is observed to be increasing, must be operating.
2. Solar indirect (modulation of cloudiness by cosmic rays, which vary inversely with solar wind strength) is much less understood if it works at all.
3. Soot, while altering forcing at different heights in the atmosphere, may or may not result in large net warming.

Since each mechanism warms in slightly different ways at different locations and times, attempts can be made to evaluate their significance in terms of how well their expected "fingerprints" are seen in the observations. For instance, well-mixed GHGs would tend to warm more at night, winter and at higher, cooler latitudes (less water vapor to complicate the picture). Soot should be confined to and downwind from where it is produced, because it is rained out in only a week or so. Solar indirect should vary with the solar cycle enough to show global temperature variations over that cycle.

What is seen? As usual, the picture is murky with mixed support for each:

### *GHG Theory*

Warming is indeed observed to be more at night and in winter than daytime and summer[116]. High latitudes do indeed see larger warming but this trend stops before one gets to the poles, which are not warming much at all. This might be due to energy being lost to melting ice, and it could be showing cooling effects of the polar ozone holes.

### *Solar Indirect*

If correlations of cloudiness with solar activity 1980 to 1995 are accurate, the total solar forcing is several times solar direct. This would allow solar forcing to account for some 75% or more of 20$^{th}$ century warming. But if this were so, the forcing would modulate climate over the 10- to 12-year solar cycle at such an amplitude as to be seen in the temperature record. (Wigley's modeling results[58] using only solar forcing show clear temperature variability over a cycle, but with GHG variations added these variations are much less obvious in better agreement with observations.)

### *Soot Forcing*

If recent calculations of the magnitude of soot forcing are correct, most of the warming in the past quarter century might be expected to stem from soot alone, but Ramanathan et al.[92] show that high levels of soot observed during INDOEX (Indian Ocean Experiment), while altering warming with altitude, had little affect on the net forcing over the oceans, which already have low albedo.

### SST/Cloudiness "Iris" Effect

Lindzen's work[114] seems to show that increased SST (sea surface temperature) inhibits growth of high-level stratiform ice clouds downwind from deep convective events due to coagulation of rain drops, rendering the upper part of the cloud relatively drier. If true, this would represent a thermostat-like negative feedback, allowing radiative cooling into space over abnormally warm oceans. However, Ramanathan et al.[115] reported results from the Central Equatorial Pacific Experiment showing just the opposite effect — increased cloudiness following increased deep convection over abnormally warm oceans. Also, a very recent publication presents data that contradicts this hypothesis and shows that such an effect, if present, would actually cause slight warming[129].

Thus, while there remain other potential explanations for the observed warming, especially in the second half of the last century, that of the AGHG seems the most likely, matching more of the observations than the others. However, as the IPCC stresses, there remain lingering uncertainties, and this is an area of continued study.

### Model Tuning

It is sometimes objected that there are so many free parameters in the GCMs that the clever modeler can simply tune them to get most any desired behavior. This shows a lack of appreciation of how free one is to "tune" the models. While it is true that some parameterizations are tuned to match observations (and it would be best to move to process models that do not require such tuning), the climate itself is so complex and chaotic that it would take an extremely large number of trial tuning runs to effect any desired outcome, and the resultant tuning would then be unlikely to be correct for the next set of studies. In addition I have cited several instances above where either outright predictions were made that were subsequently borne out, or where analysis of runs, already made for other reasons, showed agreement with observations. In these cases tuning was not a possibility.

### Summary

Attribution of observed warming in the last hundred years is largely in the realm of complex climate simulating computer codes. While leaving much to be desired, especially in their treatment of clouds and vertical distribution of water vapor, their ability to reproduce the large-scale features of observed climate gives us confidence that they can be relied upon to simulate climate change over the last century. When they are used in this way, they are unable to account for warming in the second half of the 20$^{th}$ century employing natural forcings alone. However, addition of anthropogenic greenhouse gas and aerosol forcing results in quite close agreement between models and observations. The possibility of other forcings such as soot and solar-modulated cloudiness merits continued research, but these are estimated not to be large enough to alter the IPCC's basic conclusion that human emissions are a major cause of recent warming.

Finally, adequate simulation of the last century's temperature record increases our confidence in predictions of warming in the next century with a range in temperatures depending on the climate sensitivity of the codes used.

## OTHER TRENDS (IS ANYTHING ELSE CHANGING BESIDES TEMPERATURE?)

As global warming increases might there be other indicators of climate change? For example, one might expect a more energetic weather system with increased storminess. A warmer world would increase evaporation of surface moisture, perhaps leading to increases in hurricanes, large storms and more tornadoes. As storm tracks adjust and jet streams strengthen, regional precipitation changes as well as extremes in both high and low temperatures might be experienced. At sea, especially in the North Atlantic, increased precipitation might reduce the ocean's saltiness leading to a slowing of the THC, in which cold and salty — and therefore dense — water sinks making room for warm surface water to penetrate farther north. If that circulation were to slow, Europe would actually experience cooler climate. In fact as warmer, more humid tropical air moves north and south, one might see increased snowfall actually thickening the Greenland and Antarctic ice sheets. A warmer ocean would thermally expand, increasing sea level, which might further increase due to melting of polar land ice (melting sea ice does not increase sea level). A warmer Earth would see regional changes in both flora and fauna. Forests might migrate poleward; tropical conditions could invade mid-latitudes with the possibility of spread of tropical diseases. Agriculture would experience these changes with cooler weather crops having to be moved northward and warmer weather pests having to be dealt with. The climate system's great cyclic changes such as ENSO, NAO, PDO, etc. might alter timing or character. Increased $CO_2$ would affect plant growth differentially between C3 and C4 plants (which employ different photosynthesis strategies), perhaps changing the demographics of our natural areas.

Some of these and possibly other effects might occur. But will they, by how much, how fast, and which ones? If any are likely to change dramatically, have we seen their beginnings already? Or will the warming that is predicted be small enough that outside of higher temperatures at night and during winter, little change will be experienced?

Questions like these are of great interest to society and have spawned a large amount of research as observers and modelers alike look for such changes. For better or for worse, they have appeared in the press in sometimes exaggerated forms so that every heat wave, drought and flood is seen as a result of global warming and as a portent of worse to come. This review will not look exhaustively at this subject, but will give a brief survey of what is known and being done. Good summaries of this work and its findings (most of the values quoted below come from this publication) appear in the IPCC's Third Assessment Report[116] and in[117]). The bottom line is that only modest changes have been observed and no really large changes are certain. But there is considerable concern because of what is simply not known. There are scenarios in which factors could combine to cause large or unforeseen changes.

## Storminess/Precipitation

There is evidence for increased precipitation (a few tenths to 1% per decade in the NH, less in the SH) and cloudiness (~2% per decade in the NH). The disquieting thing is that precipitation seems to be coming in the form of heavier events rather than being spread out over time. The additional moisture runs off into streams causing erosion but not substantially increasing long-term soil moisture[118,119]. Thus, one might expect increases in floods rather than increased soil moisture. There is no evidence of increased numbers of tornadoes or hurricanes. In fact, were global warming to increase El Niños over La Niñas, there might be a decrease of hurricanes because El Niños strengthen the subtropical jet, which in turn inhibits growth of hurricanes.

## Snow Cover, Sea Ice and Sea Level Change

While it is agreed that a long-term effect of global warming is melting of land ice, which will lead to significant rise in sea level, initial warming may not cause much change due to potential precipitation (snow) increases. However satellites have observed about a 10% decrease in snow cover since the late 1960s. Arctic sea ice has retreated by about the same percentage since the 1950s, but Antarctic sea ice amount remains relatively constant. Observations of sea level change are complicated by continental rebound as land masses, relieved of their burden of glacial ice, continue to rise. Regional changes in sea height also complicate measurements. Best estimates of the current rate of rise are 1.0 to 2.0 mm/year, and the expected rise in the $21^{st}$ century is ~0.5 m. However, this value is highly uncertain and could be significantly larger.

## Regional Changes in Precipitation

Perhaps the largest change in a warmer climate may not be the temperature but precipitation. As storm tracks change, regional variations are to be expected. Thus, agricultural productivity may change significantly from region to region with winners as well as losers. The resulting societal response is not a pleasant prospect. While there are some indications of such changes, the record is far too short and our understanding of natural climate variation too limited to allow more than suggestive attribution of these to global warming.

### *Changes in Cyclic Patterns such as ENSO*

Much of our climate is shaped by large-scale cyclic weather patterns. The best known of these, ENSO, alternates between cold and warm surface water in the eastern equatorial Pacific. The attendant changes in global weather are well documented as are its large economic consequences. As this pattern alternates between El Niño (warm surface water) and La Niña (cold water), North America alternates wet and dry winters between north and south regions. These alternations are about evenly distributed with a 4- to 7-year period. In addition there seems to be a lower frequency alternation (~25 to 40 years) in predominance of one extreme over the other. Most recently in the mid-1970s a multidecade period of predominating La Niña was replaced with predominating El Niño. These alternations cause considerable disruption of agriculture, cattle production, etc. Were global warming to shift ENSO into a predominance of one or the other extreme, or worse, to increase their magnitude, this would require significant societal adjustment. The 1970s switch to El Niño predominance has been suggested as just such an effect synchronized as it was with a rapid rise in global temperatures[120]. Others, however, suggest that the switch is largely natural, has happened before and is in fact the cause of a large part of recent warming. Another alternating weather pattern, the NAO (of persistent high/low pressure alternating between Iceland and the Canary Islands), strongly affects temperatures and precipitation in the Atlantic and Europe. It is in some sense synchronized with ENSO and in a similar chicken/egg fashion is caused by or adding to NH warming since the mid 1970s.

Climate scientists have only recently begun to appreciate the large influence of these coupled regional climate oscillations in climate change, much less to understand how the oscillations may be interacting with global warming or are being affected by global warming. Several fertile suggestions have been put forth regarding how these patterns might dominate climate[121,122,123]. If any of these turn out to be correct, they will have a great influence on how we understand climate change with and without global warming.

## Changes in the North Atlantic THC

Some computer models predict that, as warming increases, this vital circulation, which warms Europe, will slow[124,125]. Indeed such a slowing has been observed. However, other models see little slowing, and the observed change may be part of a natural cyclic variation. Understanding the behavior of this so-called “conveyor belt” circulation is of extreme importance since it has been linked to extremely large amplitude global climate change in the past[126]. This is all the more worrisome since such changes can be extremely rapid, having been observed to occur in only a decade or so. In fact possible linkage between warming and THC remains the obvious wild card in future climate and must be studied carefully.

## Fauna Changes

There is growing literature documenting migrations of insects, poleward extensions of ranges of birds and plants, etc. such that there is no longer any doubt of the response of living things to warming thus far. Continued warming will only increase these migrations.

## POLICY (CAN SCIENCE ASSIST PLANNING?)

As mentioned earlier, with the issue of global warming, the science of climate change has taken on the unaccustomed dimension of forming the basis for nothing less than global policy and planning regarding use of fossil fuels and other activities involving AGHGs. This creates the need for scientists to communicate definitive information to society, information that is of its very nature uncertain. Given human reliance on fossil fuels to drive economies and the acknowledged difficulty of alternative energy forms to supplant this source, society demands a level of certainty of risk that climate scientists are unaccustomed to providing. Thus, whether global change, associated with the putative warming to come, constitutes sufficient risk to warrant the necessary response (i.e., drastically reducing reliance on fossil fuels) is the nexus of the debate. And the crucial element here is the level of certainty demanded before actions are taken. An added dimension, which perhaps makes this issue unique, is that, because of the long lifetime of $CO_2$ in the atmosphere, a lead time for action is necessary. In other words, by the time we are actually experiencing unwanted effects of AGHGs, their atmospheric concentration will be already too high for any mitigation to reverse the situation in a timely manner. Thus, policymakers are being asked to formulate rather drastic plans before we know for certain the magnitude of the problem.

The challenges for policy makers are great, but correct reading of the science is daunting. A short introduction to the issues can be found in[127]. For many, the risks, not only to humans but also to the rest of the biosphere, are simply too great to be ignored, and a prudent society will initiate steps to ameliorate that risk commensurate with its understanding of the problem in a dynamic way that allows for periodic course corrections as better information becomes available. There are others, of course, who think we know enough and should take immediate and rather drastic action to reduce AGHG emissions. For others, the level of certainty at present is far from confirming that risks are large, and, given the benefits of fossil energy and the strain on growing economies that even small actions would cause, they suggest that no significant actions are called for at this time.

It is to be expected that those who have the largest economic stake in production of energy from fossil fuels (including transportation and land use) take the latter position while those concerned with the environment and rapidly increasing problems associated with population growth take the former. Because advancing scientific knowledge is strongly adversarial — healthy skepticism drives all but the most soundly based hypotheses out — the resulting scientific debate is seized upon as evidence of high uncertainty. To further confuse policymakers, the debate has been amplified and polarized by the nonscientific societal antagonists, with each side presenting its scientists and scientific arguments. From a philosophical standpoint nonscientific policy makers are faced with such strong arguments both pro and con as to conclude that scientific knowledge is relative and, in the end, values based.

To clarify and synthesize the science, the United Nations and its WMO established the IPCC, which in turn is made up of three working groups tasked with assessing:

1. Scientific basis
2. Impacts
3. Mitigation

The idea was that by bringing together the world's scientific and economic communities, a considered opinion could be arrived at which was not driven by extremes on either side of the issue. The IPCC's First Assessment Report (FAR) came out in 1990 and was immediately updated to clarify major points in 1992. Working Group I stated explicitly that, given the information at hand, it could not attribute all or even part of the observed warming to human activities. This notwithstanding, Working Groups II and III presented a bleak picture of what could happen in a warmer climate. To discuss possible action, the UN held a world conference at Rio de Janeiro in which a treaty, the so-called Rio Accords, was considered and ratified by most nations. These accords called for voluntary measures to begin the process of reducing reliance on fossil fuels. Its basic points were that developed countries would reduce their AGHG emissions to 1990 levels by the year 2000, and provide significant financial assistance to developing countries to develop alternative energy sources. The participating developing countries agreed to take significant actions to reduce the rate of increase of their emissions. This treaty was generally accepted. For example, the United States' President (G.H.W.) Bush signed the treaty and the Senate ratified it unanimously.

However, in its Second Assessment Report (SAR) in 1996 enough new information had accrued that the IPCC made the historic statement: "Despite lingering uncertainties, the balance of evidence suggests a human influence on the climate." While this was a remarkably weak statement, it caused a firestorm of reaction, which went so far as to attack the IPCC as having been taken over by global warming proponents and having acquired a political agenda. In addition, its Policy Makers Summary (which is all many people read) was criticized as not accurately reflecting the uncertainties dealt with in the main text. The follow-up to the Rio Conference, which attempted to move nations from voluntary to mandatory commitments to reduce AGHGs, was held in Kyoto, Japan. The resultant Kyoto treaty was far more controversial. It called for developed countries to reduce emissions to slightly below 1990 levels by 2015 but it placed no restraints on developing countries. This was in part because developing countries correctly pointed out that developed countries were largely in noncompliance with the Rio Accords while they (developing countries) had made considerable strides to live up to their part. In essence they were saying, "When developed countries, who have caused the problem thus far (and profited economically from it), have shown that they can meet their obligations, only then will we obligate ourselves to emissions reductions." At this point the United States, by far the largest contributor to AGHG emissions, became a holdout for, although the administration signed the treaty, the Senate, many of whose members had voted for the Rio Accords, voted unanimously that it would reject the treaty if asked to consider it. Their stated reason was lack of participation of developing countries. The current (G.W.) Bush administration went even further and simply refused to consider Kyoto. This nearly total loss of participation and leadership by the most polluting country has caused increased turmoil within the policy community, and it remains to be seen how the United States can long remain outside the mainstream of world activity on this issue.

Given the general level of agreement that global warming is real and will likely increase significantly in the coming years, it is strange to see how the critics of the anthropogenic global warming hypothesis could have succeeded in convincing legislators that the problem is nearly nonexistent.[7] In part this has to do with the fact that reducing carbon emissions affects every individual's way of life rather than a few companies as did the Montreal Accords, which mandated reduction in chlorofluorocarbons. The prospect of having to change personal habits makes the populace and its representatives only too willing to hear that, after all, the anthropogenic global warming problem is not that bad and is only made to look so by value-driven extreme environmentalists.

In its Third Assessment Report (TAR)[116], the IPCC states that we are now more certain of human involvement in global warming. Despite increased certainty the range of predicted warmings remains essentially the same as stated by FAR and SAR. However, many have felt that TAR reflects somewhat of an embattled mentality in reaction to the attacks made on SAR (causing it to perhaps minimize uncertainties in its Policy Makers Summary). Nevertheless, its findings are widely accepted as being substantially correct. Given the endorsement of TAR by National Academies of Science from most developed countries, the opponents of the anthropogenic global warming hypothesis are now taking a twofold approach. First, they are admitting that there will be warming, but they hold that instead of mitigation to reduce emissions, it is more practicable to rely on advancing technologies to adapt to the warming, which they point out will have beneficial as well as deleterious consequences. This position is actually just a veiled way of reiterating their earlier position that the warming will be small. The second approach is to admit the possibility of anthropogenic global warming but minimize it in developing policy. Thus, legislators no longer reject the influence of AGHGs on climate, but largely ignore it in refusing to limit emissions.

Currently a number of proposed actions are being discussed. A short summary of these can be found in[128].

## CONCLUSIONS

Over the past 20 years an enormous amount of study has gone into understanding climate and, in particular, how it is responding to the increasing concentrations of AGHGs. While significant uncertainties and conundra persist, it is felt that their resolution will not alter the basic conclusions put forth in this review. It is hoped that the information and references given here will be sufficient to allow the reader to make an informed decision on how well we know the elements of the anthropogenic greenhouse warming hypothesis. It is also hoped that this information makes credible the IPCC's conclusion that there is indeed a balance of evidence to suggest that humans are causing a significant portion of the observed warming. Finally it is hoped that this review will allow the reader to put into perspective arguments to the contrary.

## ACKNOWLEDGMENTS

The author is indebted to The University of California's Institute of Geophysics and Planetary Physics — branches both at Los Alamos National Laboratory and at Scripps Institute of Oceanography, UCSD — for supporting the author as a Cecil Greene Scholar during which time most of this information was brought together. He is also indebted to the following for helpful discussions and references suggested or supplied: Mike MacCracken, Tom Wigley, Kevin Trenberth, Ben Santer, Joel Norris, Jeff Severinghaus, Warren White, David Keeling, Tim Barnett, V. Ramanathan, Jim Hansen, Michael Mann, Phil Jones, Brian Tinsley, Tom Shankland, Yvonne Keller, Richard Courtney, Onar Åm, Jarl Ahlbeck, Hartwig Volz, S.A. Boehmer-Christiansen, and John Daly. Also to Rebecca Johnson for turning journal graphs into electronic ones, Juliet Padilla and Shirley Roybal for reference formatting and to Grant Heiken for reading and commenting on the manuscript. In addition there have been a rather larger number of people who have both helped and encouraged me to take on this project. To them I am also thankful.

The Los Alamos National Laboratory is operated for the U.S. Department of Energy by the University of California. The views expressed here are those of the author and do not necessarily reflect the views of these organizations.

## GLOSSARY

ACRIM Active Cavity Radiometer Irradiance Monitor: Satellite instrument used to measure Sun's energy across the solar spectrum
AGHG Anthropogenic Greenhouse Gas: Greenhouse gas attributable to human society
AMIP Atmospheric Model Intercomparison Project
ENSO El Niño–Southern Oscillation: Large-scale ocean-atmosphere interaction phenomenon that encompasses both El Niño and La Niña; includes alternating pressure patterns over much of the tropics
ERB Earth Radiation Budget
FAR IPCC's First Assessment Review (1991)
GCM Global Climate Model: Computer model used to simulate Earth's climate, consisting generally of coupled ocean-atmosphere-land-sea ice modules
GFDL Geophysical Fluid Dynamics Laboratory (NOAA)
GISP Greenland Ice Sheet Project
GISS Goddard Institute for Space Studies (NASA)
GHG Greenhouse Gas(es): Atmospheric constituents that absorb and re-emit radiation in wavelengths coincident with Earth's emission of heat
IPCC Intergovernmental Panel on Climate Change (of the United Nations World Meteorological Organization)
LIA Little Ice Age
LWR Longwave Radiation: Infrared radiation; here, radiation associated with Earth's emission of heat
MSU Microwave Sounding Unit: Satellite instrument that measures air temperature in different atmospheric layers. MSU4 and MSU2LT refer to two of these layers
MAT Marine Air Temperature: Air temperature just above the sea surface
MPI Max-Plank-Institute für Meteorologie (Germany)
MWP Medieval Warming Period
NASA US National Aeronautics and Space Administration
NCAR National Center for Atmospheric Research (sponsored by the U.S. National Science Foundation)
NH Northern Hemisphere
NAO North Atlantic Oscillation: Large-scale alternating pressure patterns in the North Atlantic sector of the atmosphere
NOAA National Oceanic and Atmospheric Administration
OLR Outgoing Longwave Radiation: Earth's emission of heat to space
PCMDI Program for Climate Model Diagnosis and Intercomparison
PDO Pacific Decadal Oscillation: Large-scale alternating pressure patterns in the Pacific sector of the atmosphere
SAR IPCC's Second Assessment Review (1996)
SH Southern Hemisphere
SST Sea Surface Temperature
SWR Shortwave Radiation: Sunlight, both direct from the Sun and scattered within the atmosphere
TAR IPCC's Third Assessment Review (2001)
THC Thermohaline Circulation: The global "conveyer belt" circulation in the oceans, in which (heavier) cold, salty water sinks, flows along the ocean bottom, rises to the surface and is returned as warmer surface water. Much of the sinking occurs in the North Atlantic
UHI Urban Heat Island: Effect of urban areas on surface temperatures (warming), associated with changes of surface color and heat capacity

UV Ultraviolet (radiation): very short-wavelength sunlight; absorbed by stratospheric ozone

And, to make the usage clear:

Stratosphere Stably stratified, relatively quiescent region of greatly reduced clouds and precipitation above the troposphere
Troposphere Lower 85% or so of the atmosphere; where "weather" happens

## ENDNOTES

[1]As mentioned above, when the two records are overlain, the *amplitude* of the satellite temperature oscillations is seen to be significantly larger than those from the surface. This is in large part due to how a moist lapse rate of temperature with altitude amplifies temperature changes at the surface. In fact for the normal moist troposphere, one would expect an amplification factor of about 1.4 at the effective altitude of the satellite observations. This amplification could be part of the reason the satellite temperatures were warmer at the onset of the record and cooler after Mt. Pinatubo. Wentz and Schabel[23] reduce the satellite temperature anomalies by a factor of 1.6 in order to directly compare them with the surface record.

[2]A third paper[24] that looks at the radiosonde record gets essentially the same trends (although it only looks at the 1958–1998 trends, which it finds to differ due to the two disagreements (1979–1981 and 1992–1997) mentioned above. It also points out that which layer warmed most depends on hemisphere — in the NH the surface warmed more; in the southern hemisphere (SH) the troposphere warmed more. It is tempting to attribute this difference to the predominance of continents in the NH.

[3]Longer term proxies for solar activity variations come from measurements of $^{10}$Be[77,78] and atmospheric $^{14}$C, which change with solar activity/solar wind strength[79,80,81]. If these are calibrated with the instrumental record, they agree fairly well with what is known about earlier NH temperature. Crowley[48] synthesizes these with Lean's proxy[53].

[4]It is of interest here that studies of the changes in angular momentum of the sun, as it rotates about a common center with Jupiter (and the other planets to a lesser extent), have shown that the timing of the sunspot cycle is closely related to the timing of changes in this angular momentum. It is as if the interplay of convection and solar dynamo results in a tendency to recycle every 10 years or so, and that it is sufficient for minute changes in angular momentum to order those changes. This study also has shown that there is an identical periodicity of 178 years in both the motion of the sun about the center of mass of the solar system, and the sunspot cycle[83]. This finding led Landscheidt[84] to develop a complicated rubric to explain most climate phenomena in terms of solar variations.

[5]We now have two solar cycles (nearly 25 years) of satellite data that show changes in solar irradiance linked to the solar cycle[82]. The variation maximum to minimum over cycle 21 is (from ERB) 1374 to 1371.5 W $m^{-2}$ or about 0.18% and from ACRIM I, about 0.1%. For comparison Lean et al. estimate that, since the Maunder Minimum, activity has increased by about 0.24%. H&S get a bit higher total amplitude. Thus, variation over a solar cycle is about 1/3 the total increase.

[6]An excellent discussion of the Earth's energy budget — radiation, evaporation, conduction, convection, etc. — can be found in Kiehl and Trenberth[104].

[7]Critics' objections are sometimes startling, given what is understood, but can perhaps be understood in light of the large uncertainties that remain. However, their uncompromising opposition seems more deeply rooted. Long interaction with them (email over 2 years) leads the author to the following conclusion. Improvements in observations and models are not likely to change their minds because, in general, they hold that it is impossible:

1. To measure the Earth's surface temperature at the surface accurately
2. To infer the Earth's past temperature from paleo-proxy methods
3. To model the climate, which is too complex and chaotic
4. To determine that increases in atmospheric carbon come from burning of fossil fuels

When considering their objections, it is well to keep these underlying assumptions in mind.
